# A latent profile analysis for teacher education students’ learning: an overview of competencies in self-regulated learning

**DOI:** 10.3389/fpsyg.2025.1527438

**Published:** 2025-04-24

**Authors:** Janina Schel, Barbara Drechsel

**Affiliations:** Department of Psychology in School and Teaching, Institute of Psychology, Otto-Friedrich-Universität, Bamberg, Germany

**Keywords:** self-regulated learning, teacher education, latent profile analysis, learning success, goal orientation

## Abstract

**Introduction:**

Especially for teacher education students, competencies in self-regulated learning are of great importance: for their own learning during their studies, as well as for the diagnosis and support of their future students. This study aims to investigate the competencies and developmental potentials (currently low-developed areas that hold the potential for improvement) of these students’ self-regulated learning processes.

**Methods:**

Data from *N* = 240 teacher education students regarding the preaction, action, and postaction parameters of the self-regulated learning process were analyzed.

**Results:**

Through latent profile analysis (LPA), five self-regulated learning profiles were extracted and labeled as follows: process-oriented competent, preaction-volitional competent, action-cognitive competent, repetitive-low reflective, and avoiding-unreflective. The profiles were validated by learning success and goal orientation. Higher-competency profiles demonstrated better learning success and more favorable goal orientations than lower-competency profiles.

**Discussion:**

The person-centered approach of this study can help develop differentiated interventions based on learning profiles to promote self-regulated learning competencies in teacher education students, ensuring that interventions can be designed as efficiently as possible. Further potentials and limitations of the approach are discussed.

## 1 Introduction

Skills in self-regulated learning are a key competence for learning success (e.g., Dent and Koenka, 2016), but also for other significant variables, such as motivation (e.g., Theobald, 2021). According to Pintrich (2000), self-regulated learning is defined as an active and constructive process in which learners set goals for their learning in addition to observing, controlling, and regulating their cognitions, motivation, and behavior concerning pre-determined goals and external circumstances. Different definitions of self-regulated learning agree that the learner takes an active role in this complex process. In this context, student teachers adopt a special role because they are responsible for much more than the success and optimization of their learning process (Lu and Wang, 2022; Baumert and Kunter, 2006). The accurate diagnostic (e.g., the assessment of learning skills and difficulties) and the individual support of their future students in their self-regulated learning process is one of the most important tasks for teachers (Klug et al., 2015; Klug et al., 2013). Accordingly, student teachers have two tasks: first, they must develop competencies in self-regulated learning themselves, and then, in the next step, understand how they can foster self-regulated learning in their students (Michalsky and Schechter, 2013). There is existing empirical evidence that own self-regulating skills of student teachers support the latter (Shahmohammadi, 2014; Michalsky and Schechter, 2013). In addition, student teachers who are successful in self-regulation can serve as role models for their students (Kramarski and Michalsky, 2009). Consequently, the support of the self-regulated learning process of prospective teachers should be a goal of teacher education. There is empirical evidence that self-regulated learning skills can be promoted within teacher education programs (Ganda and Boruchovitch, 2018), thereby enabling pre-service teachers to develop their practical competence in creating learning environments that foster their students’ self-regulated learning (Perry et al., 2006). Nevertheless, as Muwonge et al. (2020) point out, previous research has mainly focused on how teachers can foster self-regulated learning skills in their students. Only two studies that focus on a person-centered approach could be identified, that specifically examine the self-regulated learning process of student teachers themselves (Muwonge et al., 2020; Heikkilä et al., 2012). Self-regulated learning is an extremely complex construct, which includes nonlinear, discontinuous patterns that are difficult to capture with traditional statistical approaches. In contrast, person-centered approaches (e.g., latent profile analysis) are particularly suitable for disentangling the data into learning profiles. Using latent profile analysis (LPA), the present study aims to analyze the competencies as well as the development potentials (currently low-developed areas that hold the potential for improvement) of student teachers in all phases of the complex self-regulated learning process (Schmitz and Schmidt, 2007). In this way, it can be shown (a) how heterogeneous teacher education students are in their self-regulated learning skills, (b) which competencies, and (c) which deficits the individual learning profiles exhibit in the self-regulated learning process. The findings of the study can thus provide an important empirical basis for the construction of needs-based intervention programs, that can efficiently promote competencies in self-regulated learning in future research (Dörrenbächer and Perels, 2016). The results will be systematized in a learning typology to generate an overview of the special competencies and development potentials of different self-regulated learning profiles in student teachers. Furthermore, the learning typology is intended to be validated by the learning success and goal orientations.

## 2 Theoretical framework

### 2.1 Person-centered approaches to self-regulated learning

There are several studies examining self-regulated learning through person-centered approaches. Many studies focus on students in elementary or high school (e.g., Kwarikunda et al., 2022; Zheng et al., 2020; Cleary et al., 2021; Karlen, 2016; Merchie and Van Keer, 2014; Abar and Loken, 2010). However, some clear trends in self-regulated learning (e.g., Higgins et al., 2021; Li et al., 2020; Fryer and Vermunt, 2018), suggest that university students might exhibit different learning profiles. Studies on university students often come from different study disciplines (e.g., Jeong and Feldon, 2023; Hong et al., 2020; Dörrenbächer and Perels, 2016; Räisänen et al., 2016; Ning and Downing, 2015; Liu et al., 2014; Bouchet et al., 2013; Barnard-Brak et al., 2010). Here too, it is clear that students from different disciplines can vary systematically in their self-regulated learning profiles (Parpala et al., 2022). Only a few studies concentrate on teacher education students. Heikkilä et al. (2012) used latent class clustering to identify the following three self-regulated learning profiles of 213 student teachers in Finland: non-regulating (50.0%), self-directed (28.0%), and non-reflective (22.0%). Self-directed students showed the highest use of deep learning strategies, best learning success, optimism and the lowest levels of surface learning strategies and lack of regulation. Furthermore, the learners within this profile were the least stressed and exhausted. Non-regulating students were characterized by the highest scores on lack of regulation and task-avoidance, lowest on optimism and average on approaches to learning scales. This profile reported the lowest levels of well-being and interest in their studies. Non-reflective learners showed the lowest levels of deep learning strategies and task avoidance as well as average levels on surface approaches, lack of regulation and optimism. Additionally, they exhibited an average level of stress, exhaustion, and interest in their studies. Muwonge et al. (2020) used latent profile analysis to examine the self-regulated learning process of 527 teacher education students in Uganda and found three profiles: high self-regulated learners (47.8%), average self-regulated learners (39.7%), and low self-regulated learners (12.7%). High self-regulated learners demonstrate high motivation, a wide range of learning strategies, and greater learning success compared to low self-regulated learners. In contrast, the latter show low task value, low self-efficacy as well as less control of learning beliefs. In summary, both studies point to the heterogeneity of student teachers’ competencies in self-regulated learning and highlight the far-reaching effects of these competencies beyond learning success (e.g., on well-being and motivation). The authors of both studies point to the need for differentiated support approaches. However, in the variety of constructs related to self-regulated learning, previous latent profile analyses of student teachers lack a systematic framework that represents the learning process as holistically as possible. The process model of self-regulated learning (Schmitz and Schmidt, 2007) helps to systematize the individual steps that must be followed in the learning process and can be applied to typical learning situations for students (e.g., exam preparation). In this way, a learning typology based on the model could provide insights into the specific points in the self-regulated learning process where learners need support.

### 2.2 Self-regulated learning process and learning success

As previously outlined, the theoretical framework for the present study is based on the process model of self-regulated learning by Schmitz and Schmidt (2007). The model represents a further development of the process models by Schmitz and Wiese (2006) as well as Zimmerman (2000). It divides the learning process into the following three phases: preaction, action, and postaction. A single learning cycle is not clearly defined in terms of time or content within the context of the model. The steps of the process model can be undergone both when learning a single learning unit in one day and over several weeks for exam preparation (Nett and Götz, 2019). The model arranges the parameters of self-regulated learning in a process-oriented and behaviorally relevant manner along the learners’ learning cycle. The feedback loop from the post-actional phase to a new pre-actional phase is the core element of the process model and a prerequisite for the regulation of the learning process (Schmitz et al., 2011). In the following, parameters that show particularly high significance for learning success, primarily based on empirical findings (especially from meta-analyses), are selected from all three phases of the self-regulated learning process for the latent profile analysis. In this way, the recommendation to systematically select parameters for the LPA that comprehensively cover the complex process of self-regulated learning (Jeong and Feldon, 2023) is to be followed.

#### 2.2.1 Selected parameters for learning success in the preaction phase

In the preaction phase, the goals for the learning process are set, and the actual learning activities are prepared and planned. According to findings of a meta-analysis by Sitzmann and Ely (2011), goal setting (*r* = 0.37) and self-efficacy expectations (*r* = 0.29), out of 16 constructs investigated in the realm of self-regulated learning, emerge as the most effective predictors of learning success on average. Goals provide a crucial benchmark in the learning process for monitoring, evaluation, and regulation and are closely linked to the planning of the learning process, which has been identified as a metacognitive skill and a significant component of interventions aimed at promoting self-regulated learning (meta-analyses by Zhao et al., 2025; Hemmler and Ifenthaler, 2024; Theobald, 2021; Donker-Bergstra et al., 2014). A positive self-efficacy expectation as a preaction resource in the learning process is further associated with setting challenging goals, developing effective learning strategies, and maintaining high commitment and effort toward goal attainment and thus academic outcomes (e.g., Qi et al., 2024; Kocsis and Molnár, 2024). In particular, in the context of more complex or challenging tasks, preaction resources are of paramount importance.

#### 2.2.2 Selected parameters for learning success in the action phase

In the action phase, the planned learning strategies and actions are implemented with as much focus and continuity as possible (Nett and Götz, 2019). Volitional strategies are particularly relevant when building on the preaction phase. While motivation in the preaction phase is centered on goal setting and evaluation, volition refers to the processes and phenomena necessary for the concrete realization of set goals at the action level (Achtziger and Gollwitzer, 2010). Despite being discussed as a significant component of self-regulated learning, volitional strategies are seldom identified as an independent category in theories and models in the relevant research field (Heinze, 2018; Dörrenbächer and Perels, 2015). However, in the process model proposed by Schmitz and Schmidt (2007), volitional strategies are explicitly conceptualized. Justus (2017) demonstrated that volitional strategies (such as initiation control and positive self-motivation) positively impact early and consistent learning activities in virtual higher education settings, thereby contributing to students’ learning success (see also Sansone et al., 2011). Initiation control, defined as the use of an opportunity to initiate action (Kuhl, 1996), thereby favors distributed learning, as opposed to massed learning. Distributed learning is considered to be particularly conducive to sustainable quantitative and qualitative learning outcomes (Schutte et al., 2015).

Cognitive learning strategies are often part of research on learning typologies (e.g., Muwonge et al., 2020; Räisänen et al., 2016; Dörrenbächer and Perels, 2016). Cognitive learning strategies describe how specific learning content and information are handled (Perels et al., 2020). According to Perels et al. (2020), a distinction can be made between deep and surface strategies. Repeating learning content is classified as a surface strategy, while organizing, elaborating, and critically examining learning content are classified as deep strategies. Depending on the chosen strategy, learning content can be processed either superficially or more deeply during information processing. The latter is particularly beneficial for the long-term retention of knowledge and the deeper understanding of learning content (Broadbent, 2017). Hemmler and Ifenthaler (2024) as well as Zhao et al. (2025), based on their meta-analytical findings, demonstrated that cognitive learning strategies significantly correlate with learning success. This is especially true in the university learning environment, which presents students with a variety of diverse learning demands. The complex utilization of cognitive learning strategies is positively associated with academic success (e.g., Neroni et al., 2019; Richardson et al., 2012).

According to Wild and Schiefele (1994), resource-oriented and metacognitive learning strategies (e.g., monitoring, which is referred to in the following section as a part of reflection in the postaction phase) can be classified alongside cognitive learning strategies. Resource-oriented learning strategies support the learning process by creating the fundamental prerequisites of successful learning (e.g., Landmann et al., 2015). Within this context, distinctions can be made between internal, located within the learner, and external, located outside the learner, resources (Perels et al., 2020). Wild and Schiefele (1994) mention high concentration as an internal resource and a calm and structured learning environment as an external resource. In the context of online education, as in 2021 when the current data were collected, where learners have to actively create a quiet, structured learning environment themselves, this external resource appears to be of particular importance. Accordingly, a large number of students reported limitations in having a quiet and structured learning environment during online teaching (Preböck and Annen, 2021; Lörz et al., 2020). Boerner et al. (2005) demonstrated that concentration during the learning process is a highly significant predictor of learning success. Based on the findings of Aeppli (2005), learning patterns extracted from 21 investigated parameters of self-regulated learning differed most significantly in terms of their concentration ability (in virtual studies). The meta-analyses by Zhao et al. (2025) as well as Hemmler and Ifenthaler (2024) also confirm the importance of resource-oriented learning strategies for learning success.

#### 2.2.3 Selected parameters for learning success in the postaction phase

In the postaction phase, metacognitive strategies play a crucial role in regulating the individual’s learning or learning behavior through awareness of the learning process (e.g., Kaiser, 2018). According to Donker-Bergstra et al. (2014), the provision of metacognitive strategies within interventions aimed at enhancing self-regulated learning competencies significantly increases their effect sizes. This has also been observed in recent meta-analyses (Guo, 2022; Theobald, 2021). In particular, the combined use of cognitive and metacognitive learning strategies leads to successful learning by promoting the understanding and retention of complex learning content (Glogger et al., 2012). An accurate self-monitoring in the action phase is a prerequisite for a successful self-reflection in the postaction phase (Guo, 2022). Greater learning performance and mental maturity are particularly associated with the ability for self-reflection regarding individual learning behavior and related control actions (e.g., comparing actual and desired outcomes to assess learning results; Hussy and Fritz, 2018). Therefore, postaction reflection on the learning process and outcomes facilitates the regulation of further learning cycles (e.g., strategy modification; Landmann et al., 2015) and directly influences the initiation of a new preaction phase, as conceptualized in the process model by Schmitz and Schmidt (2007). Also, in recent meta-analyses, metacognitive learning strategies are among the factors that show the strongest correlation with learning achievement (Hemmler and Ifenthaler, 2024; Zhao et al., 2025).

Based on the cited findings from meta-analyses and studies, the following parameters of the phases of the self-regulated learning process (Schmitz and Schmidt, 2007), which show a significant impact on learning success, can be derived for the LPA of the present study: Goals and planning as well as self-efficacy expectation (e.g., Zhao et al., 2025; Hemmler and Ifenthaler, 2024; Qi et al., 2024; Kocsis and Molnár, 2024; Theobald, 2021; Donker-Bergstra et al., 2014; Sitzmann and Ely, 2011) in the preaction phase, volitional parameters (initiation control and positive self-motivation; e.g., Justus, 2017; Dörrenbächer and Perels, 2015; Schutte et al., 2015; Sansone et al., 2011) as well as cognitive learning strategies (organization, elaboration, critical examination and repetition; e.g., Zhao et al., 2025; Hemmler and Ifenthaler, 2024; Muwonge et al., 2020; Neroni et al., 2019; Räisänen et al., 2016; Dörrenbächer and Perels, 2016; Richardson et al., 2012) and resource oriented learning strategies (concentration and learning environment; e.g., Zhao et al., 2025; Hemmler and Ifenthaler, 2024; Preböck and Annen, 2021; Lörz et al., 2020; Aeppli, 2005; Boerner et al., 2005) in the action phase and control as well as regulation (e.g., Zhao et al., 2025; Hemmler and Ifenthaler, 2024; Guo, 2022; Theobald, 2021; Donker-Bergstra et al., 2014; Glogger et al., 2012) in the postaction phase.

### 2.3 Self-regulated learning and goal orientation

Recent meta-analyses show that cognitive, metacognitive, and resource-oriented learning strategies (Hemmler and Ifenthaler, 2024), as well as self-efficacy (Qi et al., 2024; Kocsis and Molnár, 2024), also exhibit significant correlations with motivation and goal orientations in the learning process. Three goal orientations can be distinguished (Elliot, 1999): (a) mastery goal orientation, which focuses learning behavior on the purpose of developing skills and abilities; (b) performance goal orientation, which aims to demonstrate one’s own competencies or to surpass others; and (c) performance-avoidance goal orientation, which seeks to hide supposedly or actually underdeveloped abilities. Especially mastery goal orientation shows positive correlations with competencies in self-regulated learning: it is associated with a greater use of cognitive and metacognitive learning strategies, deeper processing of learning content, and increased persistence and effort in learning (e.g., Miller et al., 2021; Lin and Wang, 2018). In contrast, performance-avoidance goal orientation, is associated with less deep processing of learning content, less metacognitive self-regulation and lower persistence and effort in learning (e.g., Miller et al., 2021; Lin and Wang, 2018). The findings regarding the importance of performance goal orientation for the engagement in exam preparation in higher education are inconsistent (e.g., Miller et al., 2021). In line, some studies find a correlation between performance goal orientation and superficial processing of learning content (e.g., Huang, 2011), while other studies associate it with deep processing of learning content, learning efforts, and learning success (e.g., Senko et al., 2013; Diseth, 2011). Significant for the adaptive effect of performance goal orientation appears to be that mastery goal orientation is also highly developed (Pintrich, 2000). The findings from Malmberg (2008) especially highlight the importance of mastery goal orientation for teacher education students. In the longitudinal study, an increase in mastery goal orientation is associated with an increase in reflective thinking, intrinsic motivation for the teaching profession, and control-expectancy beliefs as a teacher.

### 2.4 Study rationale

The self-regulated learning process is an extremely complex construct. Previous person-centered studies often differentiate only quantitatively between competencies in self-regulated learning (e.g., Muwonge et al., 2020; Vanslambrouck et al., 2019; Fryer and Vermunt, 2018; Dörrenbächer and Perels, 2016). Furthermore, they hardly focus on teacher education students, for whom competencies in self-regulated learning are assumed to be particularly central (Shahmohammadi, 2014; Michalsky and Schechter, 2013; Kramarski and Michalsky, 2009). The self-regulated learning process can be assumed to vary considerably among individuals along the parameters of self-regulated learning. The present study aims to simplify the complexity of the diverse parameters and their interrelationships in a learning typology (Von Eye and Bogat, 2006), identifying homogenous profiles with significant differences across profiles (Wang and Wang, 2019). Thereby, deeper insights into the competencies and development potentials of student teachers in self-regulated learning should be provided. For a systematic selection of parameters for the LPA that captures the self-regulated learning process as comprehensively as possible, Barnard-Brak et al. (2010) and Dörrenbächer and Perels (2016) use Zimmerman’s (2000) process model. To our knowledge, this is the first latent profile analysis based on the phases of the further developed process model of self-regulated learning by Schmitz and Schmidt (2007). In previous LPAs, a profile with overall high competencies and one with overall low competencies in self-regulated learning were typically found (e.g., Muwonge et al., 2020; Vanslambrouck et al., 2019; Fryer and Vermunt, 2018; Dörrenbächer and Perels, 2016). According to Zimmerman (2000) and Barnard-Brak et al. (2010), learners may also primarily have competencies in the pre-action or action phase, with limitations in these phases often accompanying deficits in the post-action phase. Since the self-regulated learning process of teacher education students has not yet been investigated based on a process model using a person-centered approach, specific research questions are formulated instead of specific hypotheses. This allows the following research questions regarding the number and content of expected learning profiles to be specified:

Does an LPA model show the best model fit if it includes at least the following four profiles:

A) a profile with overall high competencies in all phases of the model,
B) a profile with overall low competencies in all phases of the model,
c) a profile with primarily pre-action (and reduced postaction) competencies,
D) a profile with primarily action (and reduced postaction) competencies?

To gain initial insights into the validity of the derived learning typology, the following research questions will be explored:

E) How do the profiles differ in terms of their learning success?

In principle it is assumed that in profiles with higher competencies in the self-regulated learning process also greater learning success can be found (e.g., Muwonge et al., 2020).

F) How do the profiles differ in terms of their goal orientations?

It is generally assumed that profiles with high competencies in the self-regulated learning process primarily exhibit a mastery goal orientation and profiles with low competencies primarily show a performance-avoidance goal orientation, while previous findings on performance goal orientation are too inconsistent to derive an assumption from them (e.g., Miller et al., 2021).

## 3 Methods

### 3.1 Participants

In the winter semester 2021/2022, during a psychology lecture for teacher education students at the university, data from *N* = 240 teacher education students were collected through voluntary participation in an online survey. Accordingly, only teacher education students were examined. There were no additional inclusion or exclusion criteria, except for participation at all three measurement points of the survey in the first two weeks of the semester. *N* = 198 participants had to be eliminated due to incomplete datasets. The sample consisted of 197 (82.1%) female participants, 42 (17.5%) male participants, and 1 (0.4%) non-binary participant, with an average age of *M* = 20.18 years (*SD* = 2.75, ranging from 18 to 36 years). The participants were distributed among the teaching programs as follows: 123 (51.3%) were studying for primary school teaching, 31 (12.9%) for secondary school teaching, and 43 (17.9%) each for upper secondary school teaching and vocational education. The average number of semesters was *M* = 2.17 (*SD* = 2.56), with 161 teacher education students in their first semester. Table 1 provides a detailed overview of the sample composition, separated by gender.

**Table 1:** Detailed overview of the sample composition, separated by gender.

Total sample *N* = 240			
Gender	Female	Male	Non-binary
*n*	197	42	1
Age			
*M* (*SD*)	20.13 (2.66)	20.45 (3.17)	19.00 (0.00)
Semester			
*M* (*SD*)	2.15 (2.52)	2.29 (2.79)	1.00 (0.00)
Teaching program			
Elementary school (GS) *n*	112	11	0
Secondary school (MS/RS) *n*	23	8	0
Upper secondary school (GY) *n*	28	1	1
Vocational education *n*	34	9	0

German school system with GS, Grundschule; MS, Mittelschule; RS, Realschule; GY, Gymnasium.

### 3.2 Measurement instruments

Table 2 provides an overview of the operationalization of the empirically guided parameters selected for the LPA of the present study and the operationalization of the examined dependent variables. The scales goals and planning (six items), repetition (seven items), organization (eight items), elaboration (eight items), critical examination (eight items), concentration (six items), learning environment (six items), control (six items), and regulation (seven items) were taken from the LIST questionnaire (Wild and Schiefele, 1994), a questionnaire in German for the assessment of learning strategies of university students. The reliabilities of the scales range from a Cronbach’s *α* of 0.82 to one of 0.92, which can be interpreted as high to excellent (Boerner et al., 2005). The Cronbach’s *α* for the scales repetition (0.72; Wild and Schiefele, 1994) and control (0.73; Boerner et al., 2005) can only be interpreted as acceptable. Items were answered on a 6-point Likert scale for agreement. Only the concentration scale exhibits a counterintuitive orientation, where higher values imply lower trait expression.

**Table 2:** Overview of the operationalization of the selected parameters for the LPA.

Scale	Reference	Item
Preaction phase		
Goals and planning	LIST (Wild and Schiefele, 1994)	I formulate learning goals that I then align my learning with.
Self-efficacy expectations	SESW (Petri, 2020)	Please indicate to what extent you feel confident in organizing your exam preparations independently and responsibly.
Action phase		
Initiation control	Volitional questionnaire adapted to online teaching (Justus, 2017)	If I have decided to study, I start as soon as possible.
Positive self-motivation	Volitional questionnaire adapted to online teaching (Justus, 2017)	When processing learning content, I usually know exactly how to increase my interest in the subject when my perseverance diminishes.
Repetition	LIST (Wild and Schiefele, 1994)	I read through my notes several times in a row.
Organization	LIST (Wild and Schiefele, 1994)	I create tables, diagrams, or visual representations to have the learning material presented in a more structured manner.
Elaboration	LIST (Wild and Schiefele, 1994)	When faced with new concepts, I envision practical applications.
Critical examination	LIST (Wild and Schiefele, 1994)	I compare the advantages and disadvantages of different theoretical concepts.
Concentration	LIST (Wild and Schiefele, 1994)	While studying, I notice that my thoughts wander.
Learning environment	LIST (Wild and Schiefele, 1994)	I arrange my learning environment to minimize distractions while studying.
Postaction phase		
Control	LIST (Wild and Schiefele, 1994)	I narrate the key contents to myself to identify any gaps in my understanding.
Regulation	LIST (Wild and Schiefele, 1994)	I change my learning technique when I encounter difficulties.
Dependent variables		
Mastery goal orientation	Questionnaire on motivation regulation (Schwinger et al., 2007)	I tell myself that I should keep working to learn as much as possible for myself.
Performance goal orientation	Questionnaire on motivation regulation (Schwinger et al., 2007)	I make it clear to myself how important it is to do well on tests and exams.
Performance-avoidance goal orientation	Questionnaire on motivation regulation (Schwinger et al., 2007)	I think about how uncomfortable it would be for me to perform worse than others.
Learning success		Abitur grade

Items were used in the original version in the German language for data collection.

Self-efficacy expectations were assessed using the SESW (Petri, 2020), a German questionnaire that measures academic entry self-efficacy expectations because a large portion of the sample was in their first semester at the time of measurement. The questionnaire consists of the subscales deadlines and strategies (eight items), motivation and cognition (three items), as well as cognitive challenges (two items), with a Cronbach’s *α* of 0.88, which can be interpreted as high. Items were answered on a 5-point Likert scale for agreement.

Volitional strategies were operationalized through adapted versions (tailored for online learning; Justus, 2017) of the scales initiation control (four items; Wild et al., 1995) and positive self-motivation (five items; Kuhl and Fuhrmann, 1998). The adapted scales show a Cronbach’s *α* of 0.82 for positive self-motivation and 0.80 to 0.86 for initiation control (Justus, 2017). Therefore, overall reliability was high. Items were answered on a 5-point Likert scale for agreement.

To assess the goal orientations, the scales for mastery goal orientation (4 items), performance goal orientation (5 items), and performance-avoidance goal orientation (3 items) self-instructions during learning were used from the German version of the questionnaire on motivation regulation (Schwinger et al., 2007). The scales show the following internal consistencies: *α* = 0.71 for mastery goal orientation, *α* = 0.89 for performance goal orientation and *α* = 0.68 for performance-avoidance goal orientation. This corresponds to reliabilities ranging from adequate to high. Items were answered on a 5-point Likert scale for agreement.

As a large portion of the sample was in their first semester at the time of measurement, learning success was operationalized using the Abitur grade (grade point average of college entrance diploma). Abitur grades are inversed-coded, with smaller values indicating higher learning success and larger values indicating lower learning success. The mean Abitur grade was 2.31 with a standard deviation of 0.55 and a range from 1.0 to 3.4.

Questionnaires were used as a common and standardized method for person-centered studies to capture the parameters of the LPA and goal orientation (e.g., Muwonge et al., 2020; see also Roth et al., 2016). To reduce methodological limitations (e.g., retrospective biases) of self-reports (e.g., Rovers et al., 2019), learning strategies were additionally assessed in a situation-specific context during the completion of a concrete learning task (writing a lecture summary; see also Leopold and Leutner, 2002). The correlations between the data from the questionnaire and the data from the concrete learning task were all significant (*p* < 0.001) and ranged from *r* = 0.37 (for repetition, with all other scales > 0.51) to *r* = 0.66 (for learning environment). The convergent validities can be interpreted as satisfactory to high. Moreover, the assessment of learning success in the form of the final exam grade (Abitur) was also used as an objective measure to validate the self-report data. Only data from the original questionnaires on general learning behavior were included in the LPA.

### 3.3 Procedure

The data were collected at the beginning of the semester (first two weeks) over three lecture sessions to keep the testing effort per measurement point minimal. During the online lecture sessions, students were given 20 min to respond to the respective items in an online survey. The datasets were matched anonymously by a code that the participants were requested to generate. Participation was voluntary and legitimized by informed consent. As an incentive, a 100 Euro restaurant voucher was raffled among all participants, who took part in all three measurement points.

### 3.4 Analysis of the LPA parameters

To assess the quality of the data for the LPA, Cronbach’s *α* was calculated for the present sample as a measure of reliability: goals and planning (*α* = 0.82), self-efficacy expectation (*α* = 0.80), initiation control (*α* = 0.82), positive self-motivation (*α* = 0.73), organization (*α* = 0.74), elaboration (*α* = 0.75), critical examination (*α* = 0.81), repetition (*α* = 0.73), concentration (*α* = 0.94), learning environment (*α* = 0.73), control (*α* = 0.61), and regulation (*α* = 0.74). The reliabilities are similar to those in the validation studies (Petri, 2020; Justus, 2017; Boerner et al., 2005; Wild and Schiefele, 1994) and can be interpreted as acceptable to excellent (except for control). The range in reliability between the parameters is understandable, as the items for concentration are very homogeneous, while other parameters include learning strategies that involve heterogeneous learning activities. Additionally, a confirmatory factor analysis was conducted on the twelve parameters and resulted in an overall acceptable model fit (low CFI, but good SRMR and RMSEA): CFI = 0.760; SRMR = 0.071; and RMSEA = 0.046. In Table 3, the pattern matrix for the standardized factor loadings of the items on the LPA parameters is presented. Table 4 provides an overview of the composite reliabilities (McDonald’s *ω*), the average variances extracted (AVE) and the heterotrait-monotrait ratios of correlations (HTMT). The composite reliabilities are almost identical to the reliability estimate using Cronbach’s *α* and thus also comparable to the validation studies. The AVE values are low (< 0.50, except for initiation control and concentration), indicating a low convergent validity of the parameters. Since the composite reliabilities are all > 0.60, the predominantly relatively low AVE values can be considered acceptable overall (Habók and Magyar, 2018). Furthermore, all HTMT values are lower than 0.80, which supports the discriminant validity of the parameters. The highest HTMT value (0.78) is found for control and regulation, which is plausible, as both parameters correspond to post-actional metacognitive strategies. Overall, the determined values for estimating reliability and construct validity are similar to those of other studies on variables of self-regulated learning (e.g., Habók and Magyar, 2018).

**Table 3:** Pattern matrix of the confirmatory factor analysis with the standardized factor loadings of the items on the LPA parameters.

Item	GP	SE	IC	PS	OG	EL	CE	RP	CC	LE	CT	RG
GP1: I do not set myself any learning goals.	0.74											
GP2: I am aware of what my goals are when learning.	0.61											
GP3: I formulate learning goals that I then align my learning with.	0.68											
GP4: Before learning, I think about how I want to learn.	0.60											
GP5: I do not plan my approach to learning.	0.74											
GP6: I do not think about my goals when learning.	0.59											
SE1: Please indicate to what extent you feel confident in independently organizing your schedule for the upcoming semesters.		0.40										
SE2: …using the available time for exam preparation effectively for studying.		0.68										
SE3: …achieving good academic performance even under time pressure.		0.40										
SE4: … organizing your exam preparations independently and responsibly.		0.70										
SE5: …identifying any knowledge gaps that may arise.		0.44										
SE6: …closing any knowledge gaps that may arise.		0.65										
SE7: …meeting deadlines (for example, for submitting assignments or registering for exams).		0.48										
SE8: …not losing motivation even after minor setbacks in your studies.		0.44										
SE9: …applying your prior knowledge from school to your studies.		0.36										
SE10: …solving subject-related tasks through logical thinking.		0.29										
SE11: …learning the subject-specific methods of your field of study.		0.48										
SE12: …understanding complex subject-related concepts.		0.41										
SE13: …learning large amounts of study material.		0.58										
IC1: If I have decided to study, I start as soon as possible.			0.65									
IC2: If I have to learn something difficult, I prefer to start right away rather than postpone it.			0.77									
IC3: If I have something unpleasant to do for my studies, I get it over with quickly.			0.73									
IC4: I often put off difficult tasks for a long time.			0.80									
PS1: When processing learning content, I usually know exactly how to increase my interest in the subject when my perseverance diminishes.				0.62								
PS2: …I can even focus on the positive aspects of a difficult learning task.				0.57								
PS3: …I manage to gradually find enjoyable aspects in a learning activity that was initially unpleasant.				0.45								
PS4: …I can usually motivate myself quite well when my perseverance starts to fade.				0.64								
PS5: …I usually know how to find enjoyment again in something that is becoming boring.				0.68								
OG1: I create tables, diagrams, or visual representations to have the learning material presented in a more structured manner.					0.52							
OG2: I make short summaries of the key contents as a memory aid.					0.60							
OG3: I go through my notes and create an outline with the most important points.					0.45							
OG4: I try to organize the material in a way that makes it easier to memorize.					0.49							
OG5: I compile short summaries with the main ideas from my notes, script, or literature.					0.60							
OG6: I underline the most important parts in texts or notes.					0.44							
OG7: For larger amounts of material, I create an outline that best reflects the structure of the content.					0.51							
OG8: I compile important technical terms and definitions into my own lists.					0.49							
EL1: I try to establish connections between the content of related subjects or courses.						0.59						
EL2: When faced with new concepts, I envision practical applications.						0.61						
EL3: I try to relate new terms or theories to terms and theories I already know.						0.53						
EL4: I visualize the concepts in my mind.						0.44						
EL5: I try to mentally connect what I’ve learned with what I already know about it.						0.49						
EL6: I come up with concrete examples for specific learning content.						0.50						
EL7: I relate what I learn to my own experiences.						0.57						
EL8: I consider whether the material I’m learning is relevant to my everyday life.						0.50						
CE1: I ask myself if the text I’m currently working through is truly convincing.							0.56					
CE2: I check whether the theories, interpretations, or conclusions presented in a text are sufficiently supported and justified.							0.54					
CE3: I think about alternatives to the claims or conclusions in the learning texts.							0.70					
CE4: The material I am currently working on serves as a starting point for developing my own ideas.							0.63					
CE5: I find it very engaging to clarify contradictory statements from different texts.							0.59					
CE6: I approach most texts critically.							0.51					
CE7: I compare the advantages and disadvantages of different theoretical concepts.							0.54					
CE8: I critically evaluate what I learn.							0.60					
RP1: I memorize the learning material from texts by repeating it.								0.40				
RP2: I read through my notes several times in a row.								0.50				
RP3: I memorize key terms to better recall important content areas during the exam.								0.57				
RP4: I memorize a self-created summary with the most important technical terms.								0.62				
RP5: I read through a text and try to recite it from memory at the end of each section.								0.34				
RP6: I memorize rules, technical terms, and formulas.								0.77				
RP7: I try to memorize the learning material using scripts or other notes.								0.60				
CC1: While studying, I notice that my thoughts wander.									0.87			
CC2: I find it hard to stay focused.									0.85			
CC3: I catch myself thinking about something completely unrelated.									0.87			
CC4: I am unfocused while studying.									0.84			
CC5: When I study, I am easily distracted.									0.85			
CC6: My concentration does not last long.									0.83			
LE1: I study in a place where I can concentrate well on the material.										0.53		
LE2: When I study, I make sure that I can find everything quickly.										0.76		
LE3: I arrange my learning environment to minimize distractions while studying.										0.48		
LE4: I always sit in the same place to study.										0.27		
LE5: My workspace is organized so that I can find everything quickly.										0.78		
LE6: I keep the most important materials within reach at my workspace.										0.62		
CT1: I skip tests and learning questions at the end of a chapter.											0.27	
CT2: To identify knowledge gaps, I review the key contents without using my materials for help.											0.48	
CT3: I ask myself questions about the material to check if I’ve understood everything.											0.46	
CT4: After each section, I pause to review what I’ve learned.											0.54	
CT5: I narrate the key contents to myself to identify any gaps in my understanding.											0.50	
CT6: If the learning material includes questions or tests, I use them to assess myself.											0.48	
RG1: If I realize that I should learn something else first, I change the sequence accordingly.												0.52
RG2: I change my learning technique when I encounter difficulties.												0.58
RG3: I adjust my study plans if I realize they cannot be implemented.												0.61
RG4: If I have difficulties while studying, I change the order in which I study the different parts.												0.41
RG5: I study in the order in which the learning material is presented.												0.72
RG6: If I realize that my approach to studying is not successful, I change it.												0.62
RG7: If I realize that I misunderstood something, I go over that part again.												0.08
RG8: If I find that the learning material is structured completely differently than I thought, I reorganize my entire approach.												0.48

GP, goals and planning; SE, self-efficacy expectation; IC, initiation control; PS, positive self-motivation; OG, organization; EL, elaboration; CE, critical examination; RP, repetition; CC, concentration (inverted); LE, learning environment; CT, control; RG, regulation. Items were used in the original version in the German language for data collection.

**Table 4:** Composite reliabilities (McDonald’s ω), average variances extracted (AVE) and heterotrait-monotrait ratios of correlations (HTMT) for the LPA parameters.

Parameter	ω	AVE	HTMT
Goals and planning	0.82	0.44	0.15–0.57
Self-efficacy expectation	0.80	0.25	0.26–0.70
Initiation control	0.83	0.54	0.11–0.61
Positive self-motivation	0.73	0.35	0.16–0.70
Organization	0.74	0.27	0.13–0.68
Elaboration	0.75	0.28	0.01–0.71
Critical examining	0.81	0.34	0.06–0.71
Repetition	0.75	0.31	0.03–0.68
Concentration	0.94	0.72	0.01–0.61
Learning environment	0.75	0.36	0.08–0.51
Control	0.61	0.21	0.35–0.78
Regulation	0.74	0.29	0.39–0.78

### 3.5 Latent profile analysis

A LPA was conducted to de-mix the sample in a person-centered manner and identify homogeneous patterns in the self-regulated learning process of teacher education students (Ferguson et al., 2020). The maximum likelihood estimation method was used in MPlus (Muthén and Muthén, 2017) to compute multiple LPAs with an increasing number of classes in an iterative process. Each model (with *k* classes) was then compared to the previous models (with *k*-1 classes). Various indicators were used to assess both absolute and relative model fit to find the best class solution. Bayesian Information Criterion (BIC), Sample Size Adjusted BIC (SABIC), and Akaike’s Information Criterion (AIC) were used as absolute model fit indices (Tein et al., 2013). Lower values imply better model fit (Masyn, 2013). Vuong-Lo–Mendell–Rubin Likelihood Ratio Test (VLMR) and Bootstrap Likelihood Ratio Test (BLRT) were used as relative model fit indicators. Significant *p*-values indicate a better fit of a *k*-class model compared to a *k*-1 class model (Ferguson et al., 2020). Entropy was considered a statistical measure of certainty in classification, where higher values (optimally > 0.80) indicate better model fit (Clark, 2010). Additionally, the mean correct class assignment probabilities should be above 0.70 for each profile (Nagin, 2005). After the LPA, a MANOVA and independent samples *t*-tests were conducted to further examine the differences between the learning profiles on the investigated parameters. Goals and planning, self-efficacy expectation, initiation control, positive self-motivation, repetition, organization, elaboration, critical examination, concentration, learning environment, control, and regulation were used as variables in the LPA to encompass the parameters of all three phases of the self-regulated learning process. For validation, MANOVA was subsequently conducted with class membership as a between-subject factor to examine mean differences in learning success and goal orientations across the extracted profiles. Before conducting the MANOVA, it was verified that all statistical assumptions (e.g., independence of the measurements, homoscedasticity, multivariate normal distribution, the absence of multicollinearity) for the analysis procedure were met.

## 4 Results

### 4.1 Descriptive statistics

Table 5 shows an overview of the descriptive statistics of the parameters used in the LPA. The correlations between the parameters range from 0.00 to 0.56 (respectively −0.54). The correlations found between the learning strategies are similar to those in other studies (e.g., Muwonge et al., 2020). It should be noted that the correlations with concentration are negative, as the scale is inversely coded. In summary, all correlations are below 0.80, indicating the absence of multicollinearity.

**Table 5:** Descriptive statistics for the parameters used in the LPA.

Parameter	*M*	*SD*	GP	SE	IC	PS	RP	OG	EL	CE	CC	LE	CT
Goals and planning	4.44	1.00											
Self-efficacy expectation	4.01	0.48	0.33^**^										
Initiation control	2.96	0.91	0.43^**^	0.26^**^									
Positive self-motivation	2.43	0.51	0.35^**^	0.50^**^	0.42^**^								
Repetition	4.46	0.84	0.28^**^	0.10	0.24^**^	0.12							
Organization	4.34	0.88	0.36^**^	0.17^*^	0.22^**^	0.17^*^	0.48^**^						
Elaboration	4.07	0.85	0.13^*^	0.33^**^	0.09	0.29^**^	0.01	0.18^**^					
Critical examination	3.03	0.91	0.10	0.35^**^	0.22^**^	0.32^**^	0.05	0.12	0.56^**^				
Concentration	3.42	1.30	−0.50^**^	−0.31^**^	−0.54^**^	−0.40^**^	−0.07	−0.19^**^	0.00	−0.10			
Learning environment	4.68	0.82	0.33^**^	0.28^**^	0.33^**^	0.22^**^	0.21^**^	0.36^**^	0.16^*^	0.07	−0.29^**^		
Control	4.31	0.63	0.26^**^	0.22^**^	0.22^**^	0.29^**^	0.27^**^	0.34^**^	0.32^**^	0.26^**^	−0.21^**^	0.18^**^	
Regulation	4.48	0.63	0.33^**^	0.42^**^	0.35^**^	0.40^**^	0.32^**^	0.40^**^	0.37^**^	0.27^**^	−0.31^**^	0.35^**^	0.53^**^

GP, goals and planning; SE, self-efficacy expectation; IC, initiation control; PS, positive self-motivation; RP, repetition; OG, organization; EL, elaboration; CE, critical examination; CC, concentration (inverted); LE, learning environment; CT, control. ^*^p < 0.05, ^**^p < 0.01.

### 4.2 LPA

The results of the VLMR and BLRT provide limited information for the relative fit of the models. Specifically, using the VLMR, none of the model comparisons is significant, while the BLRT is significant for all comparisons of class solutions (see Table 6; see also Muwonge et al., 2020). Table 7 presents the information criteria for the various class solutions. In total, the indices decrease as the number of classes increases, indicating a better fit of the models with a higher number of classes. The BIC value favors a five-class solution, while the AIC and SABIC values favor a six-class solution. According to Nylund et al. (2007), the BIC is a conservative estimator that can be considered superior to the other information criteria. It is especially suitable for parsimonious models. Due to the requirements of a parsimonious class solution, the interpretability of the classes, and the manifest class size (Ferguson et al., 2020), a five-class solution was found to be superior to a six-class solution.

**Table 6:** Significance testing of relative model fits.

*p*-values	2 classes (vs. 1 class)	3 classes (vs. 2 classes)	4 classes (vs. 3 classes)	5 classes (vs. 4 classes)	6 classes (vs. 5 classes)
VLMR	0.26	0.28	0.43	0.28	0.39
BLRT	<0.001	<0.001	<0.001	<0.001	0.01

VLMR, Vuong-Lo–Mendell–Rubin Likelihood Ratio Test; BLRT, Bootstrap Likelihood Ratio Test.

**Table 7:** Comparison of absolute model fits based on the number of extracted classes.

Information criteria	2 classes	3 classes	4 classes	5 classes	6 classes
AIC	6436.59	6288.61	6235.01	6181.11	6164.48
BIC	6565.37	6462.64	6454.29	6445.64	6474.26
SABIC	6448.09	6304.16	6254.59	6204.74	6192.15

AIC, Akaike Information Criterion; BIC, Bayesian Information Criterion; SABIC, Sample Size Adjusted BIC.

**Figure 1:**
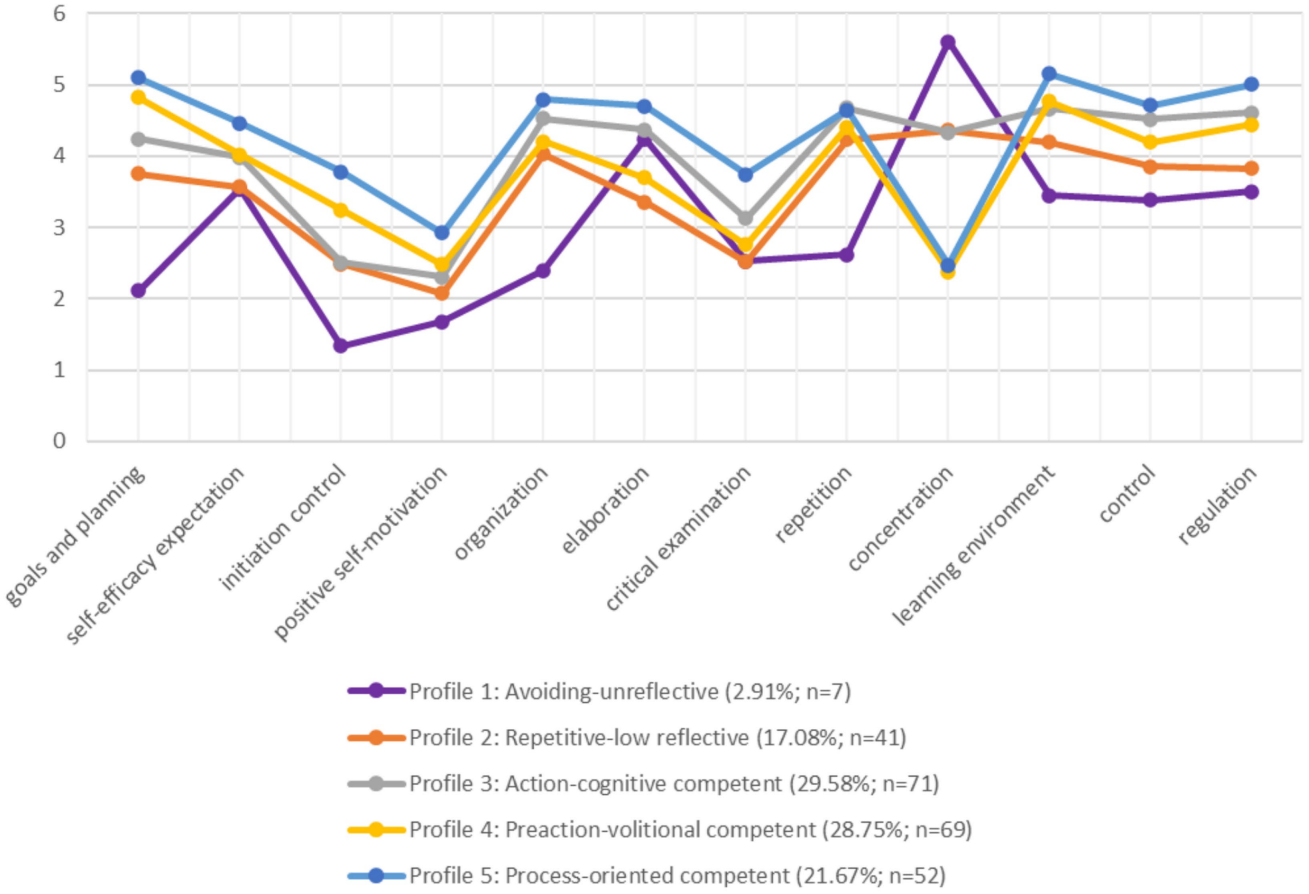
Mean parameter values, class counts and proportions of the five identified learning profiles. The concentration scale is counterintuitively polarized, so lower values are associated with higher concentration, and vice versa.

The five-class solution shows a high relative entropy of 0.83, implying good classification (Clark, 2010). All mean correct class assignment probabilities range between 0.867 and 0.997 (profile 1:0.997; profile 2:0.913; profile 3:0.893; profile 4:0.867; profile 5:0.913). This places them well above the cutoff value of 0.70 (Nagin, 2005), indicating a secure class assignment. The class counts and proportions are distributed as follows: 7 (2.91%) in profile 1; 41 (17.08%) in profile 2; 71 (29.58%) in profile 3; 69 (28.75%) in profile 4; and 52 (21.67%) in profile 5. Table 8 provides the means and standard deviations of the identified profiles for each parameter. Figure 1 visualizes the mean parameter values in the five extracted profiles. The interpretation of the identified learning profiles is provided below.

**Table 8:** Means and standard deviations of the five identified learning profiles.

Parameter	Profile 1 *n* = 7	Profile 2 *n* = 41	Profile 3 *n* = 71	Profile 4 *n* = 69	Profile 5 *n* = 52
Goals and planning	2.11 (0.38)	3.75 (0.81)	4.24 (0.85)	4.82 (0.69)	5.10 (0.81)
Self-efficacy expectation	3.54 (0.43)	3.57 (0.42)	3.99 (0.34)	4.08 (0.36)	4.47 (0.29)
Initiation control	1.34 (0.49)	2.49 (0.70)	2.51 (0.56)	3.25 (0.80)	3.78 (0.63)
Positive self-motivation	1.68 (0.51)	2.08 (0.40)	2.31 (0.41)	2.48 (0.37)	2.93 (0.39)
Organization	2.40 (0.73)	4.03 (0.75)	4.53 (0.76)	4.21 (0.75)	4.79 (0.84)
Elaboration	4.24 (1.09)	3.36 (0.56)	4.37 (0.65)	3.70 (0.64)	4.71 (0.76)
Critical examining	2.53 (0.98)	2.52 (0.66)	3.13 (0.87)	2.76 (0.70)	3.74 (0.88)
Repetition	2.62 (1.14)	4.23 (0.70)	4.68 (0.73)	4.41 (0.71)	4.65 (0.86)
Concentration	5.61 (0.52)	4.36 (0.76)	4.34 (0.86)	2.38 (0.73)	2.47 (0.71)
Learning environment	3.45 (1.41)	4.20 (0.80)	4.67 (0.78)	4.77 (0.60)	5.16 (0.60)
Control	3.38 (1.16)	3.84 (0.56)	4.52 (0.50)	4.18 (0.49)	4.70 (0.47)
Regulation	3.50 (1.07)	3.80 (0.45)	4.63 (0.40)	4.42 (0.49)	5.02 (0.41)

Standard deviations are given in parentheses after the means. The concentration scale is counterintuitively polarized.

The results indicate remarkable heterogeneity of teacher education students across the investigated parameters of self-regulated learning. All profiles demonstrate specific strengths and potential for development. Across all profiles, it is evident that the parameters of initiation control, positive self-motivation, and critical examination are comparatively low. Relatively often, in all profiles (except profile 1), learning strategies for repetition and organization are used. MANOVA with the investigated parameters as dependent variables and the learning profiles as between-subject factors indicate that the extracted profiles differed particularly in their concentration, followed by regulation, initiation control as well as self-efficacy expectation. The profiles differed the least in repetition, learning environment, organization, and critical examination. The results of the MANOVA for all parameters are summarized in Table 9.

**Table 9:** Comparisons of the self-regulated learning profiles regarding the LPA parameters.

Parameter	*df*	*F*	*p*	η_p_^2^
Goals and planning	4, 234	39.60	<0.001	0.40
Self-efficacy expectation	4, 234	48.11	<0.001	0.45
Initiation control	4, 234	48.76	<0.001	0.45
Positive self-motivation	4, 234	38.00	<0.001	0.39
Organization	4, 234	18.92	<0.001	0.24
Elaboration	4, 234	35.21	<0.001	0.38
Critical examining	4, 234	19.51	<0.001	0.25
Repetition	4, 234	13.45	<0.001	0.19
Concentration	4, 234	110.97	<0.001	0.65
Learning environment	4, 234	16.91	<0.001	0.22
Control	4, 234	24.43	<0.001	0.29
Regulation	4, 234	49.24	<0.001	0.46

Profile 5 exhibits the highest competencies in the preaction and action parameters, and postaction control and regulation are also most frequently reported. Thus, the profile demonstrates high competencies across all examined parameters of the three phases of the self-regulated learning process, in line with research question (a). Accordingly, this profile has been labeled as process-oriented competent. Nevertheless, this competent learning pattern also demonstrates development potential in the areas of positive self-motivation, initiation control, and critical examination. At 21.67%, process-oriented competent learners are less frequent in the sample than profiles 3 and 4, but more frequent than the rather deficient profiles 1 and 2.

Profiles 1 (2.91%) and 2 (17.08%) exhibit the lowest competencies in all preaction and action parameters examined. These profiles also reported the least control and regulation in the postaction phase (corresponding to research question (b)). Nevertheless, these two profiles also show qualitative differences, making the differentiation into two separate learning profiles sensible, despite the small size of profile 1. The qualitative differences between profiles 1 and 2 were tested for significance using an independent samples *t*-test (see Table 10). Profile 2 appears to be superior to profile 1, especially in planning and goal setting, initiation control, positive self-motivation, organization, repetition and finally, concentration. In contrast, profile 1 demonstrates a specific competence in the cognitive learning strategy “elaboration.” In comparison to the other profiles, profiles 1 and 2 exhibit the lowest postaction competencies. Profile 1 displays the strongest deficits overall across all phases of the self-regulated learning process. There seems to be minimal engagement with the individual’s learning process in profile 1, thereby leading to its labeling as avoiding-unreflective. Profile 2 exhibits particularly high utilization of the surface strategy of repetition, alongside comparatively high deficits in the phases of the self-regulated learning process, resulting in the labeling of this learning pattern as repetitive-low reflective.

**Table 10:** Comparison of qualitative differences between profile 1 (avoiding-unreflective; *n* = 7) and profile 2 (repetitive-low reflective; *n* = 41).

Parameter	*M*	*SD*	*df*	*t*	*p*	*d*
Goals and planning						
Profile 1	2.11	0.38	46	−5.14	<0.001	0.77
Profile 2	3.75	0.81				
Initiation control						
Profile 1	1.34	0.49		−4.23	<0.001	0.68
Profile 2	2.49	0.70				
Positive self-motivation						
Profile 1	1.68	0.51	46	−2.29	0.01	0.41
Profile 2	2.08	0.40				
Organization						
Profile 1	2.40	0.73	46	−5.47	<0.001	0.75
Profile 2	4.03	0.75				
Elaboration						
Profile 1	4.24	1.09	6.54	2.25	0.031	0.65
Profile 2	3.36	0.56				
Repetition						
Profile 1	2.62	1.14	6.79	−3.66	0.004	0.77
Profile 2	4.23	0.70				
Concentration						
Profile 1	5.61	0.52	46	4.15		0.73
Profile 2	4.36	0.76				

The concentration scale is counterintuitively polarized, so lower values are associated with higher concentration, and vice versa.

Profile 4 shows preaction competencies, high concentration, and, especially, volitional competencies. Accordingly, profile 4 was named preaction-volitional competent. However, there is no significant difference in self-efficacy expectations compared to profile 3 (*M* = 3.99, *SD* = 0.34; profile 4: *M* = 4.08, *SD* = 0.36; *t*(138) = −1.53, *p* = 0.065). There is only a significantly higher manifestation of the self-efficacy expectation sub-scale “deadlines and strategies” in profile 4 compared to profile 3 (profile 3: *M* = 4.01, *SD* = 0.40; profile 4: *M =* 4.16, *SD* = 0.38; *t*(138) = −2.31, *p* = 0.011, *d* = 0.39). The postaction competencies (control: *M =* 4.18, *SD* = 0.49 and regulation: *M* = 4.42, *SD* = 0.49) are behind those of profile 5 (control: *M* = 4.70, *SD* = 0.47, *t*(119) = −5.89, *p* = <0.001, *d* = 0.48; regulation: *M* = 5.02, *SD* = 0.41, *t*(119) = −7.15, *p* = <0.001, *d* = 0.46) and profile 3 (control: *M* = 4.52, *SD* = 0.50, *t*(138) = −4.07, *p* = <0.001, *d* = 0.50; regulation: *M* = 4.63, *SD* = 0.40, *t*(138) = −2.82, *p* = 0.003, *d* = 0.44) but ahead of those of profile 2 (control: *M* = 3.84, *SD* = 0.56, *t*(108) = 3.34, *p* = <0.001, *d* = 0.52; regulation: *M* = 3.80, *SD* = 0.45, *t*(108) = 6.64, *p* = <0.001, *d* = 0.47) and (tendentially) profile 1 (control: *M* = 3.38, *SD* = 1.16, *t*(6.22) = 1.81, *p* = 0.060; regulation: *M* = 3.50, *SD* = 1.07, *t*(6.25) = 2.24, *p* = 0.032, *d* = 0.58). Therefore, the findings are partially in line with research question (c). Profile 4 is represented in the sample about the same frequency as profile 3, at 28.75%.

The majority of participants in the sample (29.58%) belong to profile 3. Profile 3 displays competencies, particularly in the action phase, in cognitive learning strategies (organizing, elaboration, critical examination, and repetition), and, therefore, it was labeled as action-cognitive competent. In the postaction phase, profile 3 shows, after profile 5, the highest level of control (profile 3: *M* = 4.52, *SD* = 0.50; profile 5: *M =* 4.70, *SD* = 0.47; *t*(121) = −1.99, *p* = 0.024, *d* = 0.49) and regulation (profile 3: *M* = 4.63, *SD* = 0.40; profile 5: *M =* 5.02, *SD* = 0.41; *t*(121) = −5.25, *p* < 0.001, *d* = 0.49), which are also significantly higher than in profile 4 as shown above as well as in profile 2 (control: *t*(110) = 6.64, *p* < 0.001, *d* = 0.52; regulation: *t*(110) = 10.16, *p* < 0.001, *d* = 0.42) and profile 1 (control: *t*(6.23) = 2.58, *p* = 0.020, *d* = 0.58; regulation: *t*(6.17) = 2.77, *p* = 0.016, *d* = 0.49). The results support research question (d).

### 4.3 Self-regulated learning profiles and learning success

To validate the extracted learning typology, the learning outcomes of the profiles were also examined. Therefore, profiles demonstrating higher competencies in the self-regulated learning process should also exhibit greater learning success. Applying research question (e) to the discovered learning patterns, process-oriented competent learners are expected to demonstrate the highest learning success, followed by action-cognitive competent as well as preaction-volitional competent learners. The lowest learning success is anticipated among the repetitive-low reflective learners and, in particular, the avoiding-unreflective learners. The results of the ANOVA indicate a significant difference in the learning success of the self-regulated learning profiles with a small effect size, *F*(4, 234) = 2.47, *p* = 0.046, η_p_^2^ = 0.04. Table 11 provides an overview of the learning success in each profile. In line with the assumptions regarding research question (e), the process-oriented competent profile (5) demonstrates the highest learning success, followed by the preaction-volitional competent profile (4) and the action-cognitive competent profile (3), while the more deficient profiles, repetitive-low reflective (2) and, in particular, avoiding-unreflective (1), exhibit the lowest learning success. It is noteworthy that process-oriented competent learners significantly differ in their learning success only from the avoiding-unreflective and repetitive-low reflective learners in the pairwise comparisons. Significances were corrected with Tukey’s HSD (see Table 12). Tukey’s HSD was chosen because it can handle unequal sample sizes (as in the present data) and is considered a conservative measure.

**Table 11:** Descriptive statistics on learning success in the five extracted profiles.

Profiles	*n*	*M*	*SD*	*Min*	*Max*
Avoiding-unreflective (1)	7	2.59	0.34	2.1	3.2
Repetitive-low reflective (2)	41	2.41	0.55	1.0	3.3
Action-cognitive competent (3)	71	2.36	0.52	1.2	3.2
Preaction-volitional competent (4)	69	2.30	0.54	1.1	3.4
Process-oriented competent (5)	52	2.12	0.61	1.0	3.2

The Abitur grade was used as indicator for learning success.

**Table 12:** Pairwise comparisons of the self-regulated learning profiles regarding their learning success.

Profile comparison	*df*	*t*	*p*	*d*
Process-oriented competent				
vs. preaction-volitional competent	118	1.65	0.240	
vs. action-cognitive competent	121	2.37	0.918	
vs. repetitive-low reflective	91	2.33	0.011	0.58
vs. avoiding-unreflective	11.96	2.99	0.001	0.59
Preaction-volitional competent				
vs. action-cognitive competent	137	0.75	0.467	
vs. repetitive-low reflective	107	1.02	0.314	
vs. avoiding-unreflective	73	1.38	0.184	
Action-cognitive competent				
vs. repetitive-low reflective	110	0.40	0.700	
vs. avoiding-unreflective	76	1.11	0.307	
Repetitive-low reflective				
vs. avoiding-unreflective	46	0.84	0.421	

The significances of the pairwise comparisons were corrected using Tukey’s HSD.

### 4.4 Self-regulated learning profiles and goal orientations

**Figure 2:**
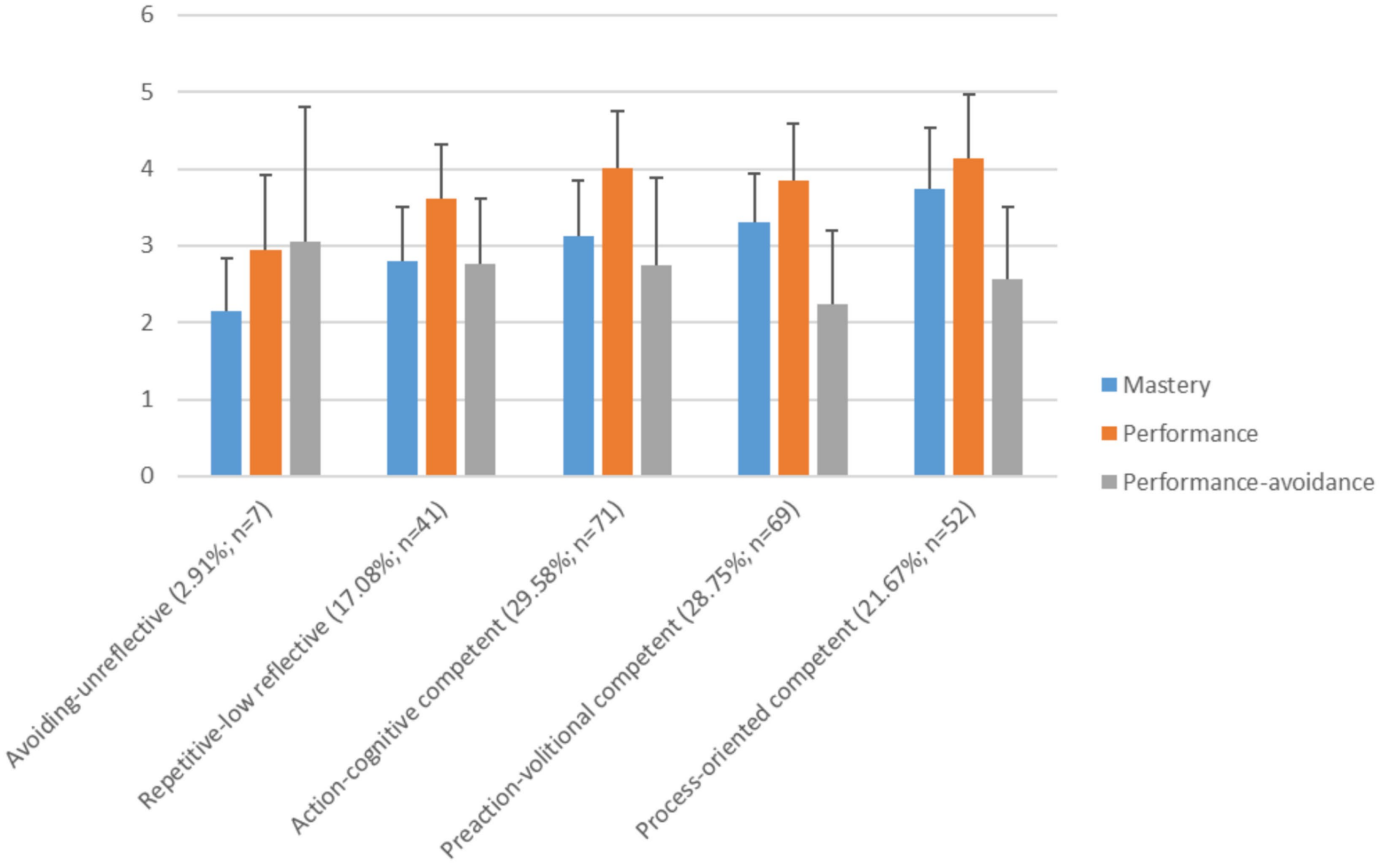
Means and standard deviations for goal orientations in the learning profiles.

To validate the learning typology beyond learning success, a MANOVA was conducted with goal orientations (mastery goal, performance goal, and performance-avoidance goal) as the dependent variables. Based on previous findings, learning profiles with higher competencies in self-regulated learning should primarily exhibit a mastery goal orientation, while profiles with deficits in self-regulated learning should show a higher performance-avoidance goal orientation. Due to the inconsistent findings regarding performance goal orientation, the profiles can only be examined exploratively for this goal orientation. Figure 2 provides an overview of the descriptive statistics (means and standard deviations) of the learning profiles in the different goal orientations. In line with research question (f), the mastery goal orientation is significantly higher in the more competent learning profiles (especially for process-oriented competent learners), with a high effect size [*F*(4, 234) = 14.84, *p* < 0.001, η_p_^2^ = 0.20]. The performance-avoidance goal orientation, however, is significantly higher in the deficient learning profiles (avoiding-unreflective and repetitive-low reflective), with a small effect size [*F*(4, 234) = 3.12, *p* = 0.016, η_p_^2^ = 0.05]. However, only the corrected pairwise comparison between preaction-volitional competent and action-cognitive competent learners is significant (*p* = 0.035, *M*_Diff_ = −0.50, 95%-CI [−0.02, −0.97]). The performance goal orientation is highest in all learning profiles (except for avoiding-unreflective) and is significantly more common in the competent profiles compared to the others, with a moderate effect size [*F*(4, 234) = 5.92, *p* < 0.001, η_p_^2^ = 0.09]. Thus, the findings are overall consistent with the assumptions regarding research question (f). Table 13 summarizes the results of the pairwise comparisons, significances were corrected using Tukey’s HSD.

**Table 13:** Pairwise comparisons of the self-regulated learning profiles regarding their goal orientations.

Profile comparison	*df*	*t*	*p*	*d*
Mastery goal orientation				
Process-oriented competent				
vs. preaction-volitional competent	119	3.18	0.013	0.72
vs. action-cognitive competent	121	4.34	<0.001	0.75
vs. repetitive-low reflective	91	5.84	<0.001	0.76
vs. avoiding-unreflective	57	4.92	<0.001	0.80
Preaction-volitional competent				
vs. action-cognitive competent	138	1.56	0.576	
vs. repetitive-low reflective	108	3.93	0.003	0.66
vs. avoiding-unreflective	74	4.59	<0.001	0.64
Action-cognitive competent				
vs. repetitive-low reflective	110	2.43	0.118	
vs. avoiding-unreflective	76	3.55	0.005	0.70
Repetitive-low reflective				
vs. avoiding-unreflective	46	2.31	0.162	
Performance goal orientation				
Process-oriented competent				
vs. preaction-volitional competent	119	2.08	0.208	
vs. action-cognitive competent	121	0.82	0.918	
vs. repetitive-low reflective	91	3.22	0.011	0.78
vs. avoiding-unreflective	57	3.49	0.001	0.85
Preaction-volitional competent				.
vs. action-cognitive competent	138	−1.45	0.619	
vs. repetitive-low reflective	108	1.55	0.578	
vs. avoiding-unreflective	74	2.93	0.029	0.77
Action-cognitive competent				
vs. repetitive-low reflective	110	2.84	0.056	
vs. avoiding-unreflective	76	3.55	0.004	0.76
Repetitive-low reflective				
vs. avoiding-unreflective	46	2.19	0.207	
Performance-avoidance goal orientation				
Process-oriented competent				
vs. preaction-volitional competent	119	1.79	0.459	
vs. action-cognitive competent	121	−0.95	0.860	
vs. repetitive-low reflective	91	−1.14	0.852	
vs. avoiding-unreflective	6.48	−0.72	0.755	
Preaction-volitional competent				
vs. action-cognitive competent	138	−2.79	0.035	1.05
vs. repetitive-low reflective	108	−2.94	0.071	
vs. avoiding-unreflective	6.36	−1.19	0.278	
Action-cognitive competent				
vs. repetitive-low reflective	103.56	−0.16	1.00	
vs. avoiding-unreflective	6.51	−0.45	0.942	
Repetitive-low reflective				
vs. avoiding-unreflective	6.47	−0.41	0.965	

The significances of the pairwise comparisons were corrected using Tukey’s HSD.

## 5 Discussion

Competencies in self-regulated learning can be assumed to be of particular importance, especially for students studying to become teachers (e.g., Lu and Wang, 2022; Michalsky and Schechter, 2013). However, there are only a few studies that attempt to provide a systematic and differentiated overview of the self-regulated learning process among teacher education students through person-centered approaches (Muwonge et al., 2020). The present study aimed to disentangle data from all three phases of the complex self-regulated learning process using a person-centered approach to gain an overview of the competencies and developmental potentials of student teachers. In line with the research questions, it was possible to (a) identify a learning profile with high competencies in all three phases of the self-regulated learning process, (b) identify two learning profiles with low competencies in all three phases of the self-regulated learning process, (c) identify a learning profile with pronounced pre-actional (and volitional) competencies, and (d) identify a learning profile with pronounced actional (primarily cognitive) competencies. These five learning profiles with quantitative and qualitative differences in self-regulated learning were labeled: process-oriented competent, preaction-volitional competent, action-cognitive competent, repetitive-low reflective, and avoiding-unreflective. Although avoiding-unreflective learners are represented only to a very small extent in the sample, significant qualitative differences between this learning pattern and repetitive-low reflective learners could be demonstrated. Consequently, the learning pattern can be meaningfully interpreted (see also Ferguson et al., 2020) and was, therefore, retained in the learning typology. Due to the high selectivity of the sample (data collection over three voluntary test points with the exclusion of 198 participants due to incomplete datasets), it can be assumed that the relatively deficient learning patterns (avoiding-unreflective as well as repetitive-low reflective) might be significantly more prevalent in practice. In summary: although the learning profile of avoiding-unreflective learners is small, a model with five profiles instead of four was chosen due to the assumed high selectivity of the sample, the interpretability of the learning profiles, and the statistical values for the absolute model fit. It should be noted that each profile displays specific strengths but also developmental potentials across the phases of the self-regulated learning process (Schmitz and Schmidt, 2007). Tus, the results of the study indicate that teacher education students demonstrate strong heterogeneity in their self-regulated learning. This demonstrates the need for differentiated approaches to promote self-regulated learning in teacher education. For an initial validation of the learning typology, significant differences in learning outcomes among the learning patterns could be identified. As assumed within the framework of research question (e), profiles showing higher competencies in the self-regulated learning process also reached greater learning success, as measured by Abitur grades. In line with the assumptions regarding research question (f), competent learning profiles also exhibited a significantly higher mastery and performance goal orientation, while deficient learning profiles showed a higher performance-avoidance goal orientation. The findings are an initial indication of the validity of the present learning typology.

Most person-centered studies examining self-regulated learning in higher education students find model solutions with three (Jeong and Feldon, 2023; Li et al., 2020; Muwonge et al., 2020; Vanslambrouck et al., 2019; Räisänen et al., 2016; Bouchet et al., 2013; Heikkilä et al., 2012) to four profiles (Fryer and Vermunt, 2018; Dörrenbächer and Perels, 2016; Ning and Downing, 2015; Liu et al., 2014). Barnard-Brak et al. (2010), who also based their parameter selection on a process model (Zimmerman, 2000), arrived at a model solution with five self-regulated learning profiles for university students in the context of online education. These can also be interpreted similarly to the self-regulated learning profiles found in the present study: Non- or minimal self-regulators, performance/reflection self-regulators, forethought-endorsing self-regulators, competent self-regulators, and super self-regulators. Forethought-endorsing self-regulators appear similar to preaction-volitional competent learners, since both profiles show competencies in the preaction phase, referred to as the forethought phase in Zimmerman’s (2000) process model. Performance/reflection self-regulators, in turn, show competencies in the action phase (or performance phase, Zimmerman, 2000), similar to action-cognitive competent learners, and also a comparatively high level of reflection in the post-action phase (or self-reflection phase, Zimmerman, 2000). In contrast to the present study, Barnard-Brak et al. (2010) found two learning profiles with high (competent self-regulators) or particularly high competencies (super self-regulators) in all phases of the self-regulated learning process. In the present study, only one corresponding learning profile was found (process-oriented competent). However, in the present study two deficient learning profiles (avoiding-unreflective and repetitive-low reflective) were found that qualitatively differ from each other. These deficient profiles are comparable to non- or minimal self-regulators according to Barnard-Brak et al. (2010). This is also in line with findings from other studies (e.g., Muwonge et al., 2020; Vanslambrouck et al., 2019; Fryer and Vermunt, 2018; Dörrenbächer and Perels, 2016; Ning and Downing, 2015) that have identified profiles of university students with consistently high competencies and low competencies in the self-regulated learning process. In further accordance with earlier learning typologies, a deficient learning pattern that primarily uses the surface learning strategy “repetition” was found in repetitive-low reflective learners (e.g., Schulz et al., 2014). In contrast to studies that primarily focus on quantitative differences in self-regulated learning competencies (e.g., Muwonge et al., 2020; Vanslambrouck et al., 2019; Fryer and Vermunt, 2018; Dörrenbächer and Perels, 2016), the systematic selection of parameters along the process model of self-regulated learning (Schmitz and Schmidt, 2007) for LPA, in particular those based on findings from meta-analyses regarding parameters that are of particular importance for learning success (e.g., Zhao et al., 2025; Hemmler and Ifenthaler, 2024; Qi et al., 2024), allows deeper insights into the specific strengths and weaknesses of the found profiles. This also enabled a further classification of learning patterns beyond “low” and “high” self-regulated learners. Therefore, action-cognitive competent learners, characterized by competencies primarily in the use of cognitive learning strategies in the action phase, and preaction-volitional competent learners, who mainly exhibit preaction and volitional competencies in the self-regulated learning process, were identified. A close relationship between preaction competencies, such as goal setting and planning of learning activities, and volitional aspects, such as initiating learning activities, can also be empirically confirmed (Pychyl and Flett, 2012). Preaction competencies (such as goal setting and planning of the learning process), as well as action competencies (for example, the use of cognitive and resource-oriented learning strategies), appear to facilitate self-regulation in the postaction phase (Zimmerman, 2000). Therefore, process-oriented competent learners also display the highest levels in the examined postaction parameters (control and regulation), while preaction-volitional competent learners, as well as action-cognitive competent learners, exhibit limitations in this area. In contrast, avoiding-unreflective, as well as repetitive-low reflective learners, demonstrate the lowest levels of control and regulation in the postaction phase. This observation means that only process-oriented competent learners, which represent only around one-fifth of the sample, possess high post-actional competencies (reflection and regulation of their learning process). These competencies, however, are considered particularly relevant for teaching students, as they are a prerequisite for optimizing their own self-regulated learning process and for designing their lessons in a way that promotes learning for their future students (Michalsky and Schechter, 2013). Although competencies in goal setting and planning, as well as control and regulation, are classified as metacognitive skills (e.g., Wild and Schiefele, 1994), action-cognitive competent learners demonstrate superiority over preaction-volitional competent learners in control and regulation (see also Barnard-Brak et al., 2010). This emphasizes the importance of competencies in cognitive learning strategies for postaction self-regulation (e.g., Glogger et al., 2012). Therefore, knowledge about cognitive learning strategies can facilitate self-regulation activities, such as changing learning strategies adaptively in a specific learning situation.

A more frequent use of learning strategies across the phases of the self-regulated learning process correlates positively with the learning success of the profiles. This is consistent with previous empirical findings in meta-analyses (e.g., Zhao et al., 2025; Hemmler and Ifenthaler, 2024), as well as in studies with person-centered approaches (e.g., Dörrenbächer and Perels, 2016; Liu et al., 2014; Valle et al., 2008). However, action-cognitive competent learners, who utilize cognitive learning strategies more frequently, especially deep strategies like “elaboration” for sustainable learning (Broadbent, 2017), exhibit tendentially lower learning success than preaction-volitional competent learners. Action-cognitive competent learners show lower initiation control compared to preaction-volitional competent learners, potentially leading to action-cognitive competent learners delaying engagement in learning activities, potentially limiting the time available for the sustainable application of cognitive learning strategies (Wäschle et al., 2014). Preaction-volitional competent learners also display a very high level of concentration, which, as an internal resource, creates favorable conditions for the application of learning strategies (see also Le, 2021). Therefore, the findings also support the importance of preaction-volitional competencies (Mccann and Turner, 2004) and internal resource-oriented learning strategies (see also Schmitz, 2001) for learning success.

The learning profiles differ most significantly from all examined dependent variables in their mastery goal orientation with a high effect size. Consistent with previous findings, the competent learning profiles (particularly process-oriented competent learners, but also preaction-volitional competent and action-cognitive competent) show a high mastery goal orientation, which is considered favorable for the self-regulated learning process (e.g., Miller et al., 2021; Lin and Wang, 2018). In contrast, deficient learning patterns (avoiding-unreflective and repetitive-low reflective) show a higher performance-avoidance goal orientation, which represents the primary goal orientation within the avoiding-unreflective profile. This is also consistent with previous findings that a performance-avoidance goal orientation is associated with deficits in the self-regulated learning process (e.g., Miller et al., 2021; Lin and Wang, 2018). The performance goal orientation is significantly higher in the competent learning profiles (process-oriented competent, preaction-volitional competent, action-cognitive competent) than in the deficient learning profiles. This could support previous findings suggesting that a high performance goal orientation combined with a high mastery goal orientation can be adaptive for the self-regulated learning process (e.g., Pintrich, 2000; see also Senko et al., 2013; Diseth, 2011). Notably, the performance goal orientation is highest in all learning profiles (except for avoiding-unreflective, which exhibit a particularly high performance-avoidance goal orientation). Especially for student teachers, a mastery goal orientation is primarily desirable, as it positively affects not only their own learning process but also their professional development as (future) educators (Malmberg, 2008). A mastery goal orientation could not only positively influence engagement in support measures aimed at optimizing the self-regulated learning process (see also Dörrenbächer and Perels, 2016) but should also be a part of such interventions (e.g., Muwonge et al., 2020).

In summary, it can be stated that all identified learning patterns show specific competencies as well as developmental potentials. In addition, over all profiles, comparatively low levels in individual estimations of the volitional parameters (initiation control as well as positive self-motivation), critical examination as a deep-level learning strategy, and distinct differences in concentration among the profiles (in line with the findings of Aeppli, 2005) are evident. These aspects can be assumed to be of particular significance, especially in less structured learning environments such as online learning settings (e.g., Zhao et al., 2025; Sansone et al., 2011; Boerner et al., 2005).

The findings of the present study allow for deeper insights into the self-regulated learning process of teacher education students, for whom competencies in self-regulated learning are of particular importance: (A) they are important role models (Kramarski and Michalsky, 2009), so their deficits in self-regulated learning (e.g., a lack of critical examination or motivation) can negatively impact the self-regulated learning process of their future students. Conversely, their competencies in this regard can serve as a resource to promote their students. Actional-cognitive competent learners as teachers, could primarily imparting resources in the area of cognitive learning strategies, but also deficits in the preactional and volitional domains. In contrast, an opposite pattern might emerge for preaction-volitional competent learners. (B) their own competencies, e.g., in cognitive and metacognitive learning strategies, can be an important prerequisite for creating a learning-supportive environment for their future students (see also Perry et al., 2006). In this context, preaction-volitional competent learners, but especially avoiding-unreflective and repetitive-low reflective learners, could show limitations. This emphasizes the importance of a differentiated consideration of the self-regulated learning process of teacher training students and the need for efficient and need oriented support approaches.

### 5.1 Practical implications

In addition to a theoretical framework, the present learning typology offers practical utility. A major advantage of person-centered approaches in the research of self-regulated learning becomes evident in the field of aptitude-treatment interactions. While learners bring a wide variety of learning prerequisites, person-centered approaches can consolidate these into a categorical factor, on the basis of which intervention recommendations can be made and evaluated (Tetzlaff et al., 2023).

The results of the present study highlight that student teachers exhibit significant heterogeneity along the self-regulated learning process. This heterogeneity can be made visible and systematized through the person-centered approach chosen within the framework of the current study. Based on the results of the study, intervention approaches can be employed to promote self-regulated learning in general, e.g., initiating learning activities earlier, as all found learning profiles show relative limitations in initiation control. A possible way to implement this could be an incentive system (e.g., Özer, 2021). At the university, students can upload weekly brief lecture summaries to a digital tool throughout the semester. The incentive is that the submitted pages will be provided as aids during the exam. This approach can help students initiate and maintain learning activities earlier in the exam preparation process (Herrmann, 2024). The derived intervention approaches can also be tailored to individual profiles, in line with resource-based counseling and support models. The adaptive use of prompts in the learning process could be a promising direction (Theobald, 2021), in order to promote self-regulated learning in a differentiated way, even in large cohorts as in teacher training programs. In this context, the mentioned incentive system can be combined with differentiated prompts to provide targeted support for students with different learning profiles. Thus, the learning profiles can be differentially supported in the application of cognitive learning strategies through cognitive prompts on their lecture summary pages (see also Saks and Leijen, 2019; Glogger et al., 2012; Hübner et al., 2010). Particularly competent learning profiles in this area (process-oriented competent and action-cognitive competent) could receive prompts that encourage critical examination as a particularly complex cognitive learning strategy, while preaction-volitional competent learners (with moderate competencies in cognitive learning strategies) could be guided toward elaboration strategies, and the rather deficient learning profiles (avoiding-unreflective and repetitive-low reflective) could be supported with less complex cognitive learning strategies, such as organizational strategies (Schel and Drechsel, 2025). The cognitive prompts could be supplemented with metacognitive prompts to encourage self-reflection in the individual learning process (Schel and Drechsel, 2025; Theobald, 2021; Donker-Bergstra et al., 2014). As part of the proposed approach, prompts from other areas of self-regulated learning could, of course, also be used: For avoiding-unreflective learners, it might be important to work initially on attention and concentration to enable the effective use of learning strategies (see also Le, 2021), action-cognitive competent learners could, in turn, benefit from prompts that support setting goals and planning the learning process (see also Daumiller and Dresel, 2019). It is noteworthy that the extraction of self-regulated learning patterns should not lead to the withholding of support approaches, a common criticism of learning typologies (Nett and Götz, 2019). Instead, the derivation of modular support measures should primarily aim to reduce the complexity of such supports for learners and to increase motivation for continuous participation in training content (Günther, 2021; Daumiller and Dresel, 2019; Davis et al., 2017). Accordingly, Dörrenbächer and Perels (2016) were able to demonstrate that students with different self-regulated learning patterns benefit differently from an intervention. In their study, students who had moderate competencies in self-regulated learning as well as students with low competencies in self-regulated learning but high motivation were the ones who primarily benefited from training to improve self-regulated learning. The study findings emphasize the importance of differentiated support measures for learners with different learning patterns. However, these findings also highlight that self-regulated learning patterns are subject to change. This is in line with the findings of Li et al. (2020), which suggest that intervention measures should be designed adaptively. However, there are currently few studies that include the longitudinal examination of learning typologies (Theobald, 2021; Schulz et al., 2014). Consequently, there is little evidence regarding differences in the dynamics of the natural development of various learning profiles throughout university studies. In particular, throughout the course of the teacher training program, the self-regulated learning process might develop differently than in other study disciplines, as self-regulated learning and teaching are central components of the curriculum.

### 5.2 Limitations

For the present data, both statistically and theoretically, a model with five profiles in total appears to be the best solution. However, the validity of this model is subject to some limitations, which will be discussed below. It should be noted that, avoiding-unreflective learners are only minimally represented in the sample, raising the question of whether this category is relevant in the learning typology (Masyn, 2013). As the data for the learning typology were collected over three measurement points (this was the only possible way because the data used were part of a larger survey), and only complete datasets were included in the analysis, it can be assumed that this is a selective sample. Therefore, the sample may consist primarily of particularly committed teacher education students who attended all three lecture sessions during which testing took place and voluntarily completed the online survey. In particular, the deficient self-regulated learning patterns (avoiding-unreflective and repetitive-low reflective) could be significantly more prevalent in practice and is retained in the analysis for theoretical reasons. Furthermore, there are clear qualitative differences between both profiles (for example, in terms of planning and goal setting, initiation control, use of repetition and organizational strategies, as well as concentration). Therefore, a five-class solution appears to be more meaningful for the interpretability of the self-regulated learning patterns compared to a four-class solution (Ferguson et al., 2020). The interpretation of the profile “avoiding-unreflective learners” should be approached with caution. Accordingly, there is a need to replicate the class solution found here in a further sample of teacher education students at a single measurement point. Reliable methods for power analyses to determine *a priori* the minimum required sample size for LPAs are still in development (Ferguson et al., 2020). However, some studies recommend that sample sizes with a minimum of 300 to 500 participants are favorable for LPA to increase statistical power and obtain robust results (e.g., Nylund et al., 2007). Accordingly, the learning typology should definitely be replicated in a larger sample (Schel and Drechsel, n.d.). The collection of data at a single measurement point would also be likely to increase the total number of complete datasets in the sample. It should also be noted that a large portion of the sample in the present study consists of primary school teaching students. However, this group is similarly represented in the total population of teaching students at the University of Bamberg (as of 2021). The gender ratio of the present sample, with an overrepresentation of female students, also reflects the overall population in the humanities at the University of Bamberg in the winter semester 2021/2022. Since there are studies suggesting a gender difference in self-regulated learning (e.g., Liu et al., 2021), the found learning typology could be more representative of female teacher education students, and therefore, it should not be interpreted as universally applicable across genders.

Another consideration for replication is that the existing data were collected during pandemic-induced online teaching. In the context of in-person teaching, the manifestations of self-regulated learning patterns could change (e.g., Vanslambrouck et al., 2019). Since online learning environments are often less structured, competencies in self-regulated learning may gain additional significance (e.g., Xu et al., 2022). A factor that should also be taken into consideration.

The assessment of self-regulated learning through questionnaires represents a common method in the field of research (Roth et al., 2016). Although validated and established questionnaires were used for the present study, limitations in the quality of the data concerning the convergent validity were observed in the present sample. Only the parameters concentration and initiation control showed good AVE values. In contrast, an acceptable model fit was observed in the confirmatory factor analysis, as well as reliabilities (Cronbach’s *α* and McDonald’s *ω*) and discriminant validities (HTMT), all of which were within the respective cutoff values. Similar patterns of statistical coefficients are also observed in other validation studies of questionnaire data on self-regulated learning (e.g., Habók and Magyar, 2018). This could be due to the fact that the parameters of self-regulated learning generally encompass heterogeneous facets of a domain (e.g., different learning activities in the area of organizational strategies) and are not independent of each other when considered as indicators of self-regulated learning. The HTMT values, as well as the correlation matrix, show that, for example, there are significant associations between the parameters control and regulation, which are both indicators of post-actional metacognition, and elaboration and critical thinking, which in turn are indicators of cognitive deep learning strategies. Nevertheless, the HTMT values and the results of the correlation analysis argue against multicollinearity of the parameters and support their discriminant validity. Since the quality of the data can overall only be rated as acceptable in terms of convergent validity, the self-regulated learning profiles based on these data in the present study should be interpreted with caution. The findings underscore the relevance of thoroughly examining the quality of the data foundation in LPA studies and also supports the need for a replication of the current learning typology, as well as a multi-method validation of it. However, questionnaires in general only inquire about knowledge of learning strategies; neither conditional knowledge nor the active application of learning strategies in everyday learning can be assessed through them (Rovers et al., 2019). Therefore, current studies advocate a multi-method approach in the assessment of self-regulated learning (Dörrenbächer-Ulrich et al., 2021). The present learning typology, like most others (e.g., Muwonge et al., 2020), relies solely on data from questionnaires. The participants’ assessments were validated based on a specific learning task, which helps avoid at least retrospective biases. However, the evaluation of the learning strategies employed in the specific learning task was also a self-report. A methodological influence could also be a reason why the relationships found between the learning profiles and goal orientations, which were all assessed through self-reports, are stronger than the relationships between the learning profiles and learning success, which was assessed objectively (Hemmler and Ifenthaler, 2024). Therefore, the self-regulated learning patterns identified here should be investigated further using other, particularly behaviorally relevant (e.g., trace data, Bernacki, 2018), methods to increase the ecological validity of the findings.

Additionally, the data on learning typology were collected cross-sectionally, making it impossible to draw conclusions about the development and dynamics of the constructs (see also Schulz et al., 2014) or to derive causal statements about the effects of learning profiles on learning success and goal orientations. In this context, it must also be considered that confounding factors (e.g., mediators and moderators, Hemmler and Ifenthaler, 2024), for example, prior knowledge (see also Simonsmeier et al., 2021) or procrastination (see also Sun et al., 2023), could affect the observed relationships. The identified self-regulated learning profiles differ significantly in their initiation control of learning activities, so procrastination could be a significant mediator of the relationship to learning success. Accordingly, the found relationships to the dependent variables should be interpreted with caution, especially for learning success, since only a small effect size could be found.

### 5.3 Future research

As previously stated, this study’s learning typology should be replicated in a larger sample for more robust results and within the context of face-to-face teaching (Schel and Drechsel, n.d.). As this study relies on self-report measures, it is important to acknowledge potential biases, such as retrospective bias and social desirability bias, which may influence participants’ responses (Rovers et al., 2019). Future research could incorporate alternative methods, such as direct observations or learning analytics, to complement self-reported data. In this line, learning products as indicators for students’ actual learning behavior according to the different self-regulated learning profiles could be analyzed to validate the learning typology using multiple methods (Schel et al., 2023; Dmoshinskaia et al., 2021; Gharib et al., 2012). The creation of learning products requires the active and situation-specific application of cognitive learning strategies (see also Callan and Cleary, 2018) and could thereby avoid the methodological limitations of self-report measures (Spörer and Brunstein, 2006). This is a potentially fruitful step toward bridging the gap from “introspection only” in questionnaires to actual learning behaviors. The deficit profile “avoiding-unreflective” reported, for example, a single resource in terms of “elaboration.” This could be verified through the analysis of elaboration strategies (e.g., deriving practical examples from theoretical content) in actively generated learning products (e.g., lecture summaries in the context of an exam preparation phase; Wuttke, 2000). If lecture summaries as learning products are collected throughout the semester, they can also capture the dynamics in the application of cognitive learning strategies (similarly to trace data approaches; Bernacki, 2018). Additional methods would be necessary to validate data on the metacognitive parameters of the learning typology. The think-aloud method and interviews could be particularly helpful in examining information about such strategies more closely (Karaca et al., 2023; Raković et al., 2022; Räisänen et al., 2016). Therefore, the planning of learning actions, as well as the monitoring of learning results and corresponding regulations (such as adjusting learning strategies), could be shown. Multi-method approaches could also help improve the inconsistent data on the quality (particularly the convergent validity) of questionnaires for measuring self-regulated learning, as found here and in some other studies (e.g., Habók and Magyar, 2018). Once the learning typology has been validated using multi-method approaches, modular intervention strategies for the targeted enhancement of different learning types could be designed and evaluated.

In this context, it is also important to examine the identified self-regulated learning profiles longitudinally (e.g., through a latent profile transition analysis; Schel and Drechsel, n.d.) in order to compare the natural development of learners within each learning profile with an intervention-supported development (Fryer and Vermunt, 2018). Additionally, it can be investigated comparatively whether teacher training students develop differently in their self-regulated learning than students from other disciplines. Since a focus of the teacher training program is on self-regulated learning and teaching, a particularly positive development of the self-regulated learning process in teacher education students would be desirable.

Moreover, the use of experimental designs should be considered to derive causal statements about the effect of the learning profiles themselves on different dependent variables, as well as about the effectiveness of interventions aimed at promoting competencies within the individual learning profiles. Finally, additional factors, such as prior knowledge and procrastination, should be included in further analyses in order to control for their influence on the observed relationships.

## 6 Conclusion

The present study is one of the first to specifically examine the self-regulated learning process of teacher education students using a person-centered approach, highlighting both quantitative and qualitative differences in the investigated self-regulated learning skills. In conclusion, the validation against learning outcomes provides an initial indication of the practical significance of the identified self-regulated learning profiles. Overall, the present study provides a structured overview of the competencies and developmental potentials of teacher education students based on the process model of self-regulated learning (Schmitz and Schmidt, 2007). It becomes evident that teacher education students exhibit significant heterogeneity in their competencies and developmental potentials, thereby requiring tailored support. This is of particular importance for teacher education students, as they have to develop competencies in self-regulated learning themselves, but also have to foster these skills in their future students. Opportunities for differentiated support in large cohorts, such as in teacher education, could include, for example, (1) semester-long incentive systems (e.g., cheat sheets; Herrmann, 2024) to encourage profiles with volitional deficits to engage in early and continuous learning activities, and (2) the use of cognitive and metacognitive prompts (e.g., in the creation of lecture summaries) to support learners in applying (differently complex) learning strategies, e.g., during university exam preparation (Schel and Drechsel, 2025). The presented learning typology appears to be the best model solution for the present data. However, it should be further validated in future research, particularly (a) with a larger sample, (b) in a longitudinal study to examine the stability and development of the learning profiles, and (c) with behaviorally related data to compensate for the methodological limitations of self-reports (Schel and Drechsel, n.d.; Theobald, 2021).

## Data Availability

The raw data supporting the conclusions of this article will be made available by the authors, without undue reservation.

## References

[ref1] Abar B. Loken E. (2010). Self-regulated learning and self-directed study in a pre-college sample. Learn. Individ. Differ. 20, 25–29. doi: 10.1016/j.lindif.2009.09.002, PMID: 20161484 PMC2794205

[ref2] Achtziger A. Gollwitzer P. M. (2010). “Motivation und Volition im Handlungsverlauf” in Motivation und Handeln. eds. Heckhausen J. Heckhausen H.. 4th ed (Berlin: Springer), 277–302.

[ref3] Aeppli J. (2005). Selbstgesteuertes Lernen von Studierenden in einem Blended-Learning- Arrangement: Lernstil-Typen, Lernerfolg und Nutzung von webbasierten Lerneinheiten. [Unpublished dissertation]. Zürich: University of Zürich.

[ref7] Barnard-Brak L. Paton V. O. Lan W. Y. (2010). Profiles in self-regulated learning in the online learning environment. Int. Rev. Res. Open Distributed Learn. 11, 61–80. doi: 10.19173/irrodl.v11i1.76

[ref8] Baumert J. Kunter M. (2006). Stichwort. Professionelle Kompetenz von Lehrkräften. Z. Erzieh. 9, 469–520. doi: 10.1007/s11618-006-0165-2

[ref9] Bernacki M. L. (2018). “Examining the cyclical, loosely sequenced, and contingent features of self- regulated learning: trace data and their analysis” in Handbook of self-regulation of learning and performance. eds. Schunk D. H. Greene J. A.. 2nd ed (Cambridge: Cambridge University Press), 370–387.

[ref10] Boerner S. Seeber G. Keller H. Beinborn P. (2005). Lernstrategien und Lernerfolg im Studium. Zeitschrift für Entwicklungspsychologie und Pädagogische Psychologie 37, 17–26. doi: 10.1026/0049-8637.37.1.17

[ref11] Bouchet F. Harley J. M. Trevors G. J. Azevedo R. (2013). Clustering and profiling students according to their interactions with an intelligent tutoring system fostering self-regulated learning. J. Educ. Data Mining 5, 104–146.

[ref12] Broadbent J. (2017). Comparing online and blended learner’s self-regulated learning strategies and academic performance. Internet High. Educ. 33, 24–32. doi: 10.1016/j.iheduc.2017.01.004

[ref13] Callan G. L. Cleary T. J. (2018). Multidimensional assessment of self-regulated learning with middle school math students. Sch. Psychol. Q. 33, 103–111. doi: 10.1037/spq0000198, PMID: 28358545

[ref14] Clark S. L. (2010). Mixture* modeling with behavioral data. Los Angeles: University of California.

[ref15] Cleary T. J. Slemp J. Pawlo E. R. (2021). Linking student self-regulated learning profiles to achievement and engagement in mathematics. Psychol. Sch. 58, 443–457. doi: 10.1002/pits.22456

[ref16] Daumiller M. Dresel M. (2019). Supporting self-regulated learning with digital media using motivational regulation and metacognitive prompts. J. Exp. Educ. 87, 161–176. doi: 10.1080/00220973.2018.1448744

[ref17] Davis D. Jivet I. Kizilcec R. F. Chen G. Hauff C. Houben G. J. (2017). “Follow the successful crowd: raising MOOC completion rates through social comparison at scale” in Proceedings of the Seventh International Learning Analytics and Knowledge Conference (New York, NY: ACM Press), 454–463.

[ref18] Dent A. L. Koenka A. C. (2016). The relation between self-regulated learning and academic achievement across childhood and adolescence: a meta-analysis. Educ. Psychol. Rev. 28, 425–474. doi: 10.1007/s10648-015-9320-8

[ref19] Diseth A. (2011). Self-efficacy, goal orientations and learning strategies as mediators between preceding and subsequent academic achievement. Learn. Individ. Differ. 21, 191–195. doi: 10.1016/j.lindif.2011.01.003

[ref20] Dmoshinskaia N. Gijlers H. de Jong T. (2021). Learning from reviewing peers’ concept maps in an inquiry context: commenting or grading, which is better? Stud. Educ. Eval. 68:100959. doi: 10.1016/j.stueduc.2020.100959

[ref21] Donker-Bergstra A. S. de Boer H. Kostons D. Dignath van Ewijk C. C. van der Werf M. P. C. (2014). Effectiveness of learning strategy instruction on academic performance: a meta-analysis. Educ. Res. Rev. 11, 1–26. doi: 10.1016/j.edurev.2013.11.002

[ref22] Dörrenbächer L. Perels F. (2015). Volition completes the puzzle: development and evaluation of an integrative trait model of self-regulated learning. Frontline Learn. Res. 3, 14–36. doi: 10.14786/flr.v3i4.179

[ref23] Dörrenbächer L. Perels F. (2016). Self-regulated learning profiles in college students: their relationship to achievement, personality, and the effectiveness of an intervention to foster self-regulated learning. Learn. Individ. Differ. 51, 229–241. doi: 10.1016/j.lindif.2016.09.015

[ref24] Dörrenbächer-Ulrich L. Weißenfels M. Russer L. Perels F. (2021). Multimethod assessment of self-regulated learning in college students: different methods for different components? Instr. Sci. 49, 137–163. doi: 10.1007/s11251-020-09533-2

[ref25] Elliot A. J. (1999). Approach and avoidance motivation and achievement goals. Educ. Psychol. 34, 169–189. doi: 10.1207/s15326985ep3403_3

[ref26] Ferguson S. L. Moore E. W. Hull D. M. (2020). Finding latent groups in observed data: a primer on latent profile analysis in Mplus for applied researchers. Int. J. Behav. Dev. 44, 458–468. doi: 10.1177/0165025419881721

[ref27] Fryer L. K. Vermunt J. D. (2018). Regulating approaches to learning: testing learning strategy convergences across a year at university. Br. J. Educ. Psychol. 88, 21–41. doi: 10.1111/bjep.12169, PMID: 28691734

[ref28] Ganda D. R. Boruchovitch E. (2018). Promoting self-regulated learning of Brazilian preservice student teachers: results of an intervention program. Front. Educ. 3, 1–12. doi: 10.3389/feduc.2018.00005, PMID: 40196160

[ref29] Gharib A. Phillips W. Mathew N. (2012). Cheat sheet or open-book? A comparison of the effects of exam types on performance, retention, and anxiety. Online Submission 2, 469–478. doi: 10.17265/2159-5542/2012.08.004

[ref30] Glogger I. Schwonke R. Holzäpfel L. Nückles M. Renkl A. (2012). Learning strategies assessed by journal writing: prediction of learning outcomes by quantity, quality, and combinations of learning strategies. J. Educ. Psychol. 104, 452–468. doi: 10.1037/a0026683

[ref31] Günther S. A. (2021). “The impact of social norms on students’ online learning behavior: insights from two randomized controlled trials” in Presentation at the LAK21: 11th international learning analytics and knowledge conference (New York, NY: ACM Press).

[ref32] Guo L. (2022). The effects of self-monitoring on strategy use and academic performance: a meta-analysis. Int. J. Educ. Res. 112:101939. doi: 10.1016/j.ijer.2022.101939

[ref33] Habók A. Magyar A. (2018). Validation of a self-regulated foreign language learning strategy questionnaire through multidimensional modelling. Front. Psychol. 9:1388. doi: 10.3389/fpsyg.2018.01388, PMID: 30127759 PMC6087767

[ref34] Heikkilä A. Lonka K. Nieminen J. Niemivirta M. (2012). Relations between teacher students’ approaches to learning, cognitive and attributional strategies, well-being, and study success. High. Educ. 64, 455–471. doi: 10.1007/s10734-012-9504-9

[ref35] Heinze D. (2018). Die Bedeutung der Volition für den Studienerfolg. Fachmedien: Springer.

[ref36] Hemmler Y. M. Ifenthaler D. (2024). Self-regulated learning strategies in continuing education: a systematic review and meta-analysis. Educ. Res. Rev. 45:100629. doi: 10.1016/j.edurev.2024.100629

[ref37] Herrmann D. (2024). “Klausur-Booklets zur Stärkung von Methodenkompetenzen und zur Reduktion von Prokrastination” in Diversität und Digitalität in der Hochschullehre. Innovative Formate in digitalen Bildungskulturen. eds. Witt T. Hermann C. Mrohs L. Brodel H. Lindner K. Maidanjuk I. (Bielefeld: transcript Verlag), 169–180.

[ref38] Higgins N. L. Rathner J. A. Frankland S. (2021). Development of self-regulated learning: a longitudinal study on academic performance in undergraduate science. Res. Sci. Technol. Educ. 41, 1242–1266. doi: 10.1080/02635143.2021.1997978, PMID: 40101104

[ref39] Hong W. Bernacki M. L. Perera H. N. (2020). A latent profile analysis of undergraduates’ achievement motivations and metacognitive behaviors, and their relations to achievement in science. J. Educ. Psychol. 112, 1409–1430. doi: 10.1037/edu0000445

[ref40] Huang C. (2011). Achievement goals and achievement emotions: a meta-analysis. Educ. Psychol. Rev. 23, 359–388. doi: 10.1007/s10648-011-9155-x

[ref41] Hübner S. Nückles M. Renkl A. (2010). Writing learning journals: instructional support to overcome learning-strategy deficits. Learn. Instr. 20, 18–29. doi: 10.1016/j.learninstruc.2008.12.001

[ref42] Hussy W. Fritz A. (2018). “Problemlösen, Planen und Metakognition” in Pädagogische Psychologie. eds. Fritz A. Hussy W. Tobinski D.. 3rd ed Stuttgart: UTB, 129–154.

[ref43] Jeong S. Feldon D. F. (2023). Changes in self-regulated learning profiles during an undergraduate peer-based intervention: a latent profile transition analysis. Learn. Instr. 83:101710. doi: 10.1016/j.learninstruc.2022.101710

[ref44] Justus X. (2017). Selbstregulation im virtuellen Studium: Volitionale Regulation, Lernzeit und Lernstrategien in Online-Seminaren [Unpublished dissertation]. Regensburg: Universität Regensburg.

[ref45] Kaiser R. (2018). “Das Konzept Metakognition” in Metakognition die neue Didaktik: Metakognitiv fundiertes Lehren und Lernen ist Grundbildung. eds. Kaiser A. Kaiser R. Lambert A. (Göttingen: Vandenhoeck & Ruprecht), 31–68.

[ref46] Karaca M. Bektas O. Celikkiran T. (2023). An examination of the self-regulation for science learning of middle school students with different achievement levels. Psychol. Sch. 60, 511–540. doi: 10.1002/pits.22776

[ref47] Karlen Y. (2016). Differences in students’ metacognitive strategy knowledge, motivation, and strategy use: a typology of self-regulated learners. J. Educ. Res. 109, 253–265. doi: 10.1080/00220671.2014.942895

[ref48] Klug J. Bruder S. Kelava A. Spiel C. Schmitz B. (2013). Diagnostic competence of teachers: a process model that accounts for diagnosing learning behavior tested by means of a case scenario. Teach. Teach. Educ. 30, 38–46. doi: 10.1016/j.tate.2012.10.004

[ref49] Klug J. Bruder S. Schmitz B. (2015). Which variables predict teachers’ diagnostic competence when diagnosing students’ learning behavior at different stages of a teacher’s career? Teachers Teach. 22, 461–484. doi: 10.1080/13540602.2015.1082729

[ref50] Kocsis Á. Molnár G. (2024). Factors influencing academic performance and dropout rates in higher education. Oxf. Rev. Educ., 1–19. doi: 10.1080/03054985.2024.2316616

[ref51] Kramarski B. Michalsky T. (2009). Investigating preservice teachers’ professional growth in self-regulated learning environments. J. Educ. Psychol. 101, 161–175. doi: 10.1037/a0013101

[ref52] Kuhl J. (1996). Who controls whom when “I control myself”? Psychol. Inq. 7, 61–68. doi: 10.1207/s15327965pli0701_12

[ref53] Kuhl J. Fuhrmann A. (1998). “Decomposing self-regulation and self-control: the volitional components inventory” in Motivation and self-regulation across the life span. eds. Heckhausen J. Dweck C. S.. 1st ed (Cambridge: Cambridge University Press), 15–49.

[ref54] Kwarikunda D. Schiefele U. Muwonge C. M. Ssenyonga J. (2022). Profiles of learners based on their cognitive and metacognitive learning strategy use: occurrence and relations with gender, intrinsic motivation, and perceived autonomy support. Human. Soc. Sci. Commun. 9, 1–12. doi: 10.1057/s41599-022-01322-1

[ref55] Landmann M. Perels F. Otto B. Schnick-Vollmer K. Schmitz B. (2015). “Selbstregulation und selbstreguliertes Lernen” in Pädagogische Psychologie. eds. Wild E. Möller J. (Berlin: Springer), 45–65.

[ref56] Le H. V. (2021). An investigation into factors affecting concentration of university students. J. English Lang. Teach. Appl. Linguist. 3, 7–12. doi: 10.32996/jeltal.2021.3.6.2

[ref57] Leopold C. Leutner D. (2002). Der Einsatz von Lernstrategien in einer konkreten Lernsituation bei Schülern unterschiedlicher Jahrgangsstufen. Zeitschrift für Pädagogik 45, 240–258. doi: 10.25656/01:3950

[ref58] Li S. Chen G. Xing W. Zheng J. Xie C. (2020). Longitudinal clustering of students’ self-regulated learning behaviors in engineering design. Comput. Educ. 153:103899. doi: 10.1016/j.compedu.2020.103899

[ref59] Lin X. Wang C. H. (2018). Achievement goal orientations and self–regulated learning strategies of adult and traditional learners. New Horizons Adult Educ. Human Resource Dev. 30, 5–22. doi: 10.1002/nha3.20229

[ref60] Liu X. He W. Zhao L. Hong J. C. (2021). Gender differences in self-regulated online learning during the COVID-19 lockdown. Front. Psychol. 12:752131. doi: 10.3389/fpsyg.2021.752131, PMID: 34603169 PMC8481384

[ref61] Liu W. C. Wang C. K. J. Kee Y. H. Koh C. Lim B. S. C. Chua L. (2014). College students’ motivation and learning strategies profiles and academic achievement: a self-determination theory approach. Educ. Psychol. 34, 338–353. doi: 10.1080/01443410.2013.785067

[ref62] Lörz M. Marczuk A. Zimmer L. Multrus F. Buchholz S. (2020). Studieren unter Corona-Bedingungen: Studierende bewerten das erste Digitalsemester. DZHW Brief 5, 1–8. doi: 10.34878/2020.05.dzhw_brief

[ref63] Lu H. Wang Y. (2022). The effects of different interventions on self-regulated learning of pre-service teachers in a blended academic course. Comput. Educ. 180:104444. doi: 10.1016/j.compedu.2022.104444

[ref64] Malmberg L. E. (2008). Student teachers' achievement goal orientations during teacher studies: antecedents, correlates and outcomes. Learn. Instr. 18, 438–452. doi: 10.1016/j.learninstruc.2008.06.003

[ref65] Masyn K. E. (2013). “Latent class analysis and finite mixture modeling” in The Oxford handbook of quantitative methods. ed. Little T. (Oxford: Oxford University Press), 551–611.

[ref66] Mccann E. J. Turner J. E. (2004). Increasing student learning through volitional control. Teachers College Record 106, 1695–1714. doi: 10.1111/j.1467-9620.2004.00401.x

[ref67] Merchie E. Van Keer H. (2014). Using on-line and off-line measures to explore fifth and sixth graders’ text-learning strategies and schematizing skills. Learn. Individ. Differ. 32, 193–203. doi: 10.1016/j.lindif.2014.03.012

[ref68] Michalsky T. Schechter C. (2013). Preservice teachers’ capacity to teach self-regulated learning: integrating learning from problems and learning from successes. Teach. Teach. Educ. 30, 60–73. doi: 10.1016/j.tate.2012.10.009

[ref69] Miller A. L. Fassett K. T. Palmer D. L. (2021). Achievement goal orientation: a predictor of student engagement in higher education. Motiv. Emot. 45, 327–344. doi: 10.1007/s11031-021-09881-7

[ref70] Muthén L. K. Muthén B. O. (2017). Mplus user’s guide. 8th Edn. Los Angeles, CA: Muthén & Muthén.

[ref71] Muwonge C. M. Ssenyonga J. Kibedi H. Schiefele U. (2020). Use of self-regulated learning strategies among teacher education students: a latent profile analysis. Soc. Sci. Human. Open 2:100037. doi: 10.1016/j.ssaho.2020.100037

[ref72] Nagin D. (2005). Group-based modeling of development. Cambridge, MA: Harvard University Press.

[ref73] Neroni J. Meijs C. Gijselaers H. J. M. Kirschner P. A. de Groot R. H. M. (2019). Learning strategies and academic performance in distance education. Learn. Individ. Differ. 73, 1–7. doi: 10.1016/j.lindif.2019.04.007

[ref74] Nett U. E. Götz T. (2019). “Selbstreguliertes Lernen” in Psychologie für den Lehrberuf, 9_4. eds. Urhahne D. Dresel M. (Berlin: Springer), 67–84.

[ref75] Ning H. K. Downing K. (2015). A latent profile analysis of university students’ self-regulated learning strategies. Stud. High. Educ. 40, 1328–1346. doi: 10.1080/03075079.2014.880832

[ref76] Nylund K. L. Asparouhov T. Muthén B. O. (2007). Deciding on the number of classes in latent class analysis and growth mixture modeling: a Monte Carlo simulation study. Struct. Equ. Model. Multidiscip. J. 14, 535–569. doi: 10.1080/10705510701575396

[ref77] Özer S. (2021). A convergent parallel mixed-method research into the use of the cheat sheet in teacher education: state test anxiety, exam scores and opinions of prospective teachers. Turk. Online J. Educ. Technol. 20, 101–113.

[ref78] Parpala A. Mattsson M. Herrmann K. J. Bager-Elsborg A. Hailikari T. (2022). Detecting the variability in student learning in different disciplines – a person-oriented approach. Scand. J. Educ. Res. 66, 1020–1037. doi: 10.1080/00313831.2021.1958256

[ref79] Perels F. Dörrenbächer-Ulrich L. Landmann M. Otto B. Schnick-Vollmer K. Schmitz B. (2020). “Selbstregulation und selbstreguliertes Lernen” in Pädagogische Psychologie. eds. Wild E. Möller J.. 3rd ed (Berlin: Springer), 45–66.

[ref80] Perry N. E. Phillips L. Hutchinson L. (2006). Mentoring student teachers to support self-regulated learning. Elem. Sch. J. 106, 237–254. doi: 10.1086/501485

[ref81] Petri P. S. (2020). Skala zur Erfassung der Studieneinstiegsselbstwirksamkeit (SESW-Skala). Zusammenstellung Sozialwissenschaftlicher Items und Skalen (ZIS). doi: 10.6102/zis274

[ref82] Pintrich P. R. (2000). “The role of goal orientation in self-regulated learning” in Handbook of self-regulated learning. eds. Boekaerts M. Pintrich P. R. Zeidner M. (Cambridge, MA: Academic Press), 451–502.

[ref83] Preböck T. Annen S. (2021). Online-Lehre im ‹Corona-Semester› aus Studierendensicht. Medienpädagogik 40, 157–176. doi: 10.21240/mpaed/40/2021.11.15.X

[ref84] Pychyl T. A. Flett G. L. (2012). Procrastination and self-regulatory failure: an introduction to the special issue. J. Ration. Emot. Cogn. Behav. Ther. 30, 203–212. doi: 10.1007/s10942-012-0149-5

[ref85] Qi B. Ma L. Wang X. (2024). Using meta-analytic path analysis to examine mechanisms relating students’ perceived feedback, motivation, self-efficacy, and academic performance. Learn. Motiv. 88:102059. doi: 10.1016/j.lmot.2024.102059

[ref86] Räisänen M. Postareff L. Lindblom-Ylänne S. (2016). University students’ self- and co-regulation of learning and processes of understanding: a person-oriented approach. Learn. Individ. Differ. 47, 281–288. doi: 10.1016/j.lindif.2016.01.006

[ref87] Raković M. Bernacki M. L. Greene J. A. Plumley R. D. Hogan K. A. Gates K. M.. (2022). Examining the critical role of evaluation and adaptation in self-regulated learning. Contemp. Educ. Psychol. 68:102027. doi: 10.1016/j.cedpsych.2021.102027

[ref88] Richardson M. Abraham C. Bond R. (2012). Psychological correlates of university students’ academic performance: a systematic review and meta-analysis. Psychol. Bull. 138, 353–387. doi: 10.1037/a0026838, PMID: 22352812

[ref89] Roth A. Ogrin S. Schmitz B. (2016). Assessing self-regulated learning in higher education: a systematic literature review of self-report instruments. Educ. Assess. Eval. Account. 28, 225–250. doi: 10.1007/s11092-015-9229-2

[ref90] Rovers S. F. E. Clarebout G. Savelberg H. H. C. M. de Bruin A. B. H. van Merriënboer J. J. G. (2019). Granularity matters: comparing different ways of measuring self-regulated learning. Metacogn. Learn. 14, 1–19. doi: 10.1007/s11409-019-09188-6

[ref91] Saks K. Leijen Ä. (2019). The efficiency of prompts when supporting learner use of cognitive and metacognitive strategies. Comput. Assist. Lang. Learn. 32, 1–16. doi: 10.1080/09588221.2018.1459729, PMID: 40101104

[ref92] Sansone C. Fraughton T. Zachary J. L. Butner J. Heiner C. (2011). Self-regulation of motivation when learning online: the importance of who, why and how. Educ. Technol. Res. Dev. 59, 199–212. doi: 10.1007/s11423-011-9193-6

[ref6] Schel J. Drechsel B. (n.d.). Replicability and stability of self-regulated learning profiles ofteacher education students. 10.1016/j.actpsy.2026.107619 42594780

[ref5] Schel J. Drechsel B. (2025). Validierung einer prompt-gestützten Intervention zur differenzierten Förderung selbstregulierten Lernens Lehramtsstudierender in der universitären Prüfungsvorbereitung. Posterpräsentation auf der 12. Tagung der Gesellschaft für Empirische Bildungsforschung (GEBF) Bildung als Schlüssel für gesellschaftliche Herausforderungen - interdisziplinäre Beiträge aus der Bildungsforschung, Mannheim.

[ref4] Schel J. Oberst L. Pensel M. Paetsch J. Drechsel B. (2023). Entwicklung und Validierung eines Instruments zur Erfassung selbstregulierten Lernens in Lernprodukten. Lehrerbildung auf dem Prüfstand, 16, 229–251. doi: 10.62350/GHBH7658

[ref93] Schmitz B. (2001). Self-Monitoring zur Unterstützung des Transfers einer Schulung in Selbstregulation für Studierende. Eine prozessanalytische Untersuchung. Zeitschrift für Pädagogische Psychologie 15, 181–197. doi: 10.1024/1010-0652.15.34.181

[ref94] Schmitz B. Klug J. Schmidt M. (2011). “Assessing self-regulated learning using diary measures with university students” in Handbook of self-regulation of learning and performance. eds. Zimmerman B. J. Schunk D. H. (New York: Routledge), 251–266.

[ref95] Schmitz B. Schmidt M. (2007). “Einführung in die Selbstregulation” in Selbstregulation erfolgreich fördern. Praxisnahe Trainingsprogramme für effektives Lernen. eds. Landmann M. Schmitz B. (Stuttgart: Kohlhammer), 9–19.

[ref96] Schmitz B. Wiese B. S. (2006). New perspectives for the evaluation of training sessions in self-regulated learning: time-series analyses of diary data. Contemp. Educ. Psychol. 31, 64–96. doi: 10.1016/j.cedpsych.2005.02.002

[ref97] Schulz F. Zehner F. Schindler C. Prenzel M. (2014). Prüfen und Lernen im Studium: Erste Schritte zur Untersuchung von Prüfungsanforderungen & Lerntypen. Beiträge zur Hochschulforschung 36, 34–58.

[ref98] Schutte G. M. Duhon G. J. Solomon B. G. Poncy B. C. Moore K. Story B. (2015). A comparative analysis of massed vs. distributed practice on basic math fact fluency growth rates. J. Sch. Psychol. 53, 149–159. doi: 10.1016/j.jsp.2014.12.003, PMID: 25746824

[ref99] Schwinger M. von der Laden T. Spinath B. (2007). Strategien zur Motivationsregulation und ihre Erfassung. Zeitschrift für Entwicklungspsychologie und Pädagogische Psychologie 39, 57–69. doi: 10.1026/0049-8637.39.2.57

[ref100] Senko C. Durik A. M. Patel L. Lovejoy C. M. Valentiner D. (2013). Performance-approach goal effects on achievement under low versus high challenge conditions. Learn. Instr. 23, 60–68. doi: 10.1016/j.learninstruc.2012.05.006

[ref101] Shahmohammadi N. (2014). Review on the impact of teachers’ behaviour on students’ self-regulation. Procedia. Soc. Behav. Sci. 114, 130–135. doi: 10.1016/j.sbspro.2013.12.672

[ref102] Simonsmeier B. A. Flaig M. Deiglmayr A. Schalk L. Schneider M. (2021). Domain-specific prior knowledge and learning: a meta-analysis. Educ. Psychol. 57, 31–54. doi: 10.1080/00461520.2021.1939700

[ref103] Sitzmann T. Ely K. (2011). A meta-analysis of self-regulated learning in work-related training and educational attainment: what we know and where we need to go. Psychol. Bull. 137, 421–442. doi: 10.1037/a0022777, PMID: 21401218

[ref104] Spörer N. Brunstein J. C. (2006). Erfassung selbstregulierten Lernens mit Selbstberichtsverfahren. Zeitschrift für Pädagogische Psychologie 20, 147–160. doi: 10.1024/1010-0652.20.3.147

[ref105] Sun T. Huynh R. Kim J.-E. (2023). Academic procrastination as a mediator between learning environment and academic performance. Proc. Human Factors Ergon. Society Ann. Meet. 67, 1578–1582. doi: 10.1177/21695067231192638

[ref106] Tein J. Y. Coxe S. Cham H. (2013). Statistical power to detect the correct number of classes in latent profile analysis. Struct. Equ. Model. Multidiscip. J. 20, 640–657. doi: 10.1080/10705511.2013.824781 PMC3904803 24489457

[ref107] Tetzlaff L. Schmitterer A. Hartmann U. Brod G. (2023). Modeling interactions between multivariate learner characteristics and interventions: a person-centered approach. Educ. Psychol. Rev. 35:112. doi: 10.1007/s10648-023-09830-5

[ref108] Theobald M. (2021). Self-regulated learning training programs enhance university students’ academic performance, self-regulated learning strategies, and motivation: a meta-analysis. Contemp. Educ. Psychol. 66:101976. doi: 10.1016/j.cedpsych.2021.101976

[ref109] Valle A. Núñez J. C. Cabanach R. G. González-Pienda J. A. Rodríguez S. Rosário P.. (2008). Self-regulated profiles and academic achievement. Psicothema 20, 724–731. 18940075

[ref110] Vanslambrouck S. Zhu C. Pynoo B. Lombaerts K. Tondeur J. Scherer R. (2019). A latent profile analysis of adult students’ online self-regulation in blended learning environments. Comput. Hum. Behav. 99, 126–136. doi: 10.1016/j.chb.2019.05.021

[ref111] Von Eye A. Bogat G. A. (2006). Person-oriented and variable-oriented research: concepts, results, and development. Merrill-Palmer Q. 52, 390–420. doi: 10.1353/mpq.2006.0032

[ref112] Wang J. Wang X. (2019). Structural equation modeling: Applications using Mplus. Hoboken, NJ: John Wiley and Sons.

[ref113] Wäschle K. Allgaier A. Lachner A. Fink S. Nückles M. (2014). Procrastination and self-efficacy: tracing vicious and virtuous circles in self-regulated learning. Learn. Instr. 29, 103–114. doi: 10.1016/j.learninstruc.2013.09.005

[ref114] Wild K.-P. Krapp A. Schiefele U. Lewalter D. Schreyer I. (1995). “Dokumentation und Analyse der Fragebogenverfahren und Tests” in Berichte aus dem DFG-Projekt "Bedingungen und Auswirkungen berufsspezifischer Lernmotivation" (Universität der Bundeswehr: München), 2.

[ref115] Wild K. P. Schiefele U. (1994). Lernstrategien im Studium. Ergebnisse zur Faktorstruktur und Reliabilität eines neuen Fragebogens. Zeitschrift für Differentielle und Diagnostische Psychologie 15, 185–200.

[ref116] Wuttke E. (2000). Lernstrategien im Lernprozess. Z. Erzieh. 3, 97–110. doi: 10.1007/s11618-000-0007-6

[ref117] Xu Z. Zhao Y. Zhang B. Liew J. Kogut A. (2022). A meta-analysis of the efficacy of self-regulated learning interventions on academic achievement in online and blended environments in K-12 and higher education. Behav. Inform. Technol. 42, 2911–2931. doi: 10.1080/0144929X.2022.2151935, PMID: 40101104

[ref118] Zhao Y. Li Y. Ma S. Xu Z. Zhang B. (2025). A meta-analysis of the correlation between self-regulated learning strategies and academic performance in online and blended learning environments. Comput. Educ. 230:105279. doi: 10.1016/j.compedu.2025.105279

[ref119] Zheng J. Xing W. Zhu G. Chen G. Zhao H. Xie C. (2020). Profiling self-regulation behaviors in STEM learning of engineering design. Comput. Educ. 143:103669. doi: 10.1016/j.compedu.2019.103669

[ref120] Zimmerman B. J. (2000). “Attaining self-regulation: a social cognitive perspective” in Handbook of self-regulation. eds. Boekaerts M. Pintrich P. Zeidner M. (Cambridge, MA: Academic Press), 13–39.

